# Magnesium Status and Ca/Mg Ratios in a Series of Children and Adolescents with Chronic Diseases

**DOI:** 10.3390/nu14142941

**Published:** 2022-07-18

**Authors:** Marlene Fabiola Escobedo-Monge, Enrique Barrado, Joaquín Parodi-Román, María Antonieta Escobedo-Monge, María Carmen Torres-Hinojal, José Manuel Marugán-Miguelsanz

**Affiliations:** 1 Faculty of Medicine, Valladolid University, Avenida Ramón y Cajal, 7, 47005 Valladolid, Spain; mctorresh@telefonica.net; 2 Department of Analytical Chemistry, Science Faculty, Campus Miguel Delibes, University of Valladolid, Calle Paseo de Belén, 7, 47011 Valladolid, Spain; ebarrado@qa.uva.es; 3 Science Faculty, Cadiz University, Paseo de Carlos III, 28, 11003 Cádiz, Spain; joaquin_parodi@yahoo.es; 4 Department of Chemistry, Science Faculty, University of Burgos, Plaza Misael Bañuelos sn, 09001 Burgos, Spain; antoitalia777@gmail.com; 5 Department of Pediatrics, Faculty of Medicine, Valladolid University, Avenida Ramón y Cajal, 7, 47005 Valladolid, Spain; jmmarugan@telefonica.net; 6 Section of Gastroenterology and Pediatric Nutrition, University Clinical Hospital of Valladolid, Avenida Ramón y Cajal, 3, 47003 Valladolid, Spain

**Keywords:** hypomagnesemia, hypermagnesemia, hypercalcemia, dietary magnesium intake, subclinical magnesium deficiency

## Abstract

Magnesium (Mg) is an essential divalent cation involved in various enzymatic reactions that regulate vital biological functions. The main goal was to evaluate Mg status and its association with nutritional indicators in 78 children and adolescents with chronic diseases. We assessed anthropometric, biochemical, diet, body composition, and bone densitometry valuations. Serum Mg and Ca levels were determined using the standardized method and diet calcium (Ca) and Mg consumption by a prospective 72 h diet survey. Mean serum Ca (9.9 mg/dL), Mg (2.08 mg/dL) dietary Ca (102% DRI: Dietary Reference Intake), and Mg intake (105% DRI) were normal. A total of 45% had hypomagnesemia, 12% had hypermagnesemia, and 26% and 24% had inadequate and high Mg intake, respectively. Only 6% of patients had poor Mg intake and hypomagnesemia, and 54% and 90% of our series had an elevated serum Ca/Mg ratio > 4.70 (mean 4.79) and a low Ca/Mg intake ratio < 1.70 (mean 1.06), respectively. Both Ca/Mg ratios were linked with the risk of developing other chronic conditions such as cardiovascular disease, type 2 diabetes, syndrome metabolic, and even several cancers. Therefore, 79% of children and adolescents with chronic diseases were at elevated risk of having abnormal Mg status and developing other chronic illnesses.

## 1. Introduction

Over the past fifty years, chronic health conditions and disabilities among children and youth have steadily risen, primarily from four classes of frequent conditions: asthma, obesity, mental health conditions, and neurodevelopmental disorders [1]. A chronic illness or medical condition is a health problem that lasts three months or more, affects a child’s regular activities, and requires frequent hospitalizations, home health care, and (or) extensive medical care [2]. These chronic diseases (lasting for years or even a lifetime) are the result of genetic (inherited) conditions, environmental factors, or a combination of both [3]. Previous epidemiological studies suggest that approximately one out of four children have a chronic disease [4], with prevalence estimates ranging from 10% to 30% [5]. In the United States, more than 40% of school-aged children and adolescents have at least one chronic health condition [6]. In addition, the impact of risk factors increases throughout life [7].

Calcium (Ca), magnesium (Mg), phosphorus (P), and vitamin D are essential nutrients for human bone development and vital functions in cellular energy metabolism. Not only are Ca, Mg, and P crucial for bone health, but also optimizing cardiac, respiratory, and neurological performance [8]. Ca is indispensable for the stability of the cell cytoskeleton and in the activity of intracellular enzyme regulation, participating in neuronal conduction through ion channels [9]. Mg is considered to be an indispensable nutrient for the management of muscle contraction, blood pressure, insulin metabolism, cardiac excitability, vasomotor tone, nerve transmission, and neuromuscular conduction [10]. The role of Mg, as a natural Ca antagonist, glutamate NMDA receptor blocker, vasodilator, antioxidant, and anti-inflammatory agent, provides therapeutic benefits [11]. Abnormal Mg level can affect DNA/RNA stabilization, enzyme activities, ion channel function, and cell protection against oxidative stress, contributing to pathological conditions [12,13]. Both aging and Mg deficiency are associated with excessive production of oxygen-derived free radicals and low-grade inflammation [14].

Chronic Mg deficits (low intake and serum levels) increase the yield of free radicals involved in the development of various chronic age-related disorders [15]. For this reason, deficient Mg status may be at least one of the pathophysiological factors that could help explain the intricate relationships between the inflammatory state and oxidative stress with the ageing process and many age-related diseases [16], including atherosclerosis [17], cardiovascular diseases (CVD), cardiac arrhythmias, hypertension (HTN) [18] and stroke [19], cardio-metabolic syndrome (CMS), coronary heart or artery disease (CHD or CAD) [13] and sudden cardiac death, alterations in lipid metabolism, insulin resistance (IR), syndrome metabolic (MetS) [20], type 2 diabetes mellitus (T2D) [21], airways constrictive syndromes and asthma [22], depression, stress-related conditions and psychiatric disorders, Alzheimer’s disease (AD) and other dementia syndromes, muscular diseases (muscle pain, chronic fatigue, and fibromyalgia), osteoporosis [23], bone fragility [15], chronic kidney disease (CKD) [24], neurodegenerative diseases, sarcopenia, frailty [22], and even cancer [15,22] (colorectal and others) [25].

The main factors that contribute to these diseases’ appearance are related to people’s diet and physical activity [26]. Deficient mineral status, most significantly of Ca, potassium (K), Mg, zinc (Zn), iron (Fe), and manganese (Mn) are associated with an impaired immune function and elevated chronic disease risks [27]. Low Mg intakes coupled with high Ca intakes and high calcium-to-magnesium (Ca/Mg) intake ratio can increase the risk of CVD, MetS, and even several cancers [28]. An imbalance of Ca and Mg in the blood may result in several clinical complications, including MetS, diabetes mellitus (DM), HTA, and CAD [29]. Low serum Mg levels or high serum Ca levels can pathologically contribute to CVD risk. In addition, serum Ca/Mg ratios may be more characteristic of homeostasis than measurements of serum Mg [30]. An adequate diet balances between Ca (milk products, eggs, fish, leafy vegetables, seeds, and nuts) [31] and Mg (cereals, nuts, seeds cocoa, soybeans, spinach, marine vegetables, and tomatoes) [32] are needed to reduce the risk of these non-communicable diseases (NCDs) [33,34].

The chronically ill pediatric population differs significantly from the chronically sick adult population in the causes of morbidity and Mg status. Nevertheless, there are few studies on Mg in healthy children and adolescents [35], and even fewer with chronic diseases, such as chronic kidney disease (CKD) [36], sickle cell disease (SCD) [37], autism spectrum [38], cystic fibrosis (CF) [39], DM [40] and other chronic conditions, and even fewer studies on Ca/Mg ratios. For this reason and to improve the knowledge about the state of Mg at these ages, we hypothesized whether an abnormal serum Mg status is prevalent in a series of chronically ill children and teenagers. Therefore, the main aim of this study was to evaluate the serum and dietary Mg intake, the Ca/Mg ratios, and their association with nutritional indicators in a series of children and adolescents with chronic diseases. 

## 2. Materials and Methods

### 2.1. Study Site, Design, and Participants

**Figure 1:**
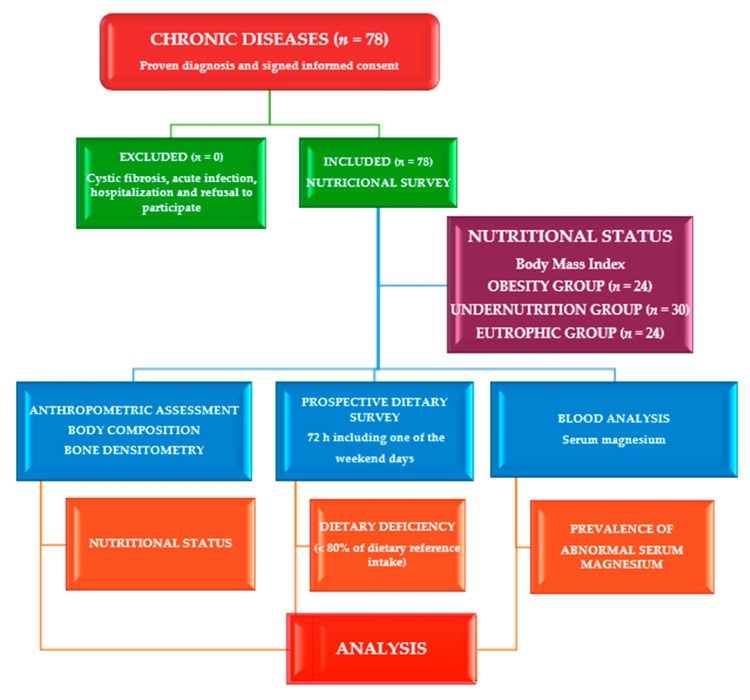
Flowchart of the study conducted on children and adolescents with chronic disease (*n* = 78).

The design of this cross-sectional and comparative study (Figure 1) was previously published in children and adolescents with chronic diseases [41,42]. We performed this study in the Nutrition Unit of the Pediatrics Service at the University Clinical Hospital in Valladolid, Spain. The sample was obtained from the number of cases evaluated in the Nutrition Unit over 18 months. Eligible subjects were selected by systematic sampling. Children under 19 years of age with a history of chronic disease were the inclusion criteria for this study. Chronic illnesses include undernutrition (unknown cause), syndromic diseases, encephalopathies, KD or disorders, hyperlipidemia, insulin-dependent diabetes mellitus (IDDM), and eating disorders. All patients underwent anthropometric, biochemical, and dietary evaluations to assess the serum and dietary Mg intake, Ca/Mg ratios, and their relationship with other nutritional indicators. Patients with CF [43] and acute infection and those who were hospitalized or refused to participate were excluded. The time of chronic diseases was shown in months. Participants were classified by age group according to Tanner stages in children and adolescents, between children ≤ 5 years and participants > 5 years, and by nutritional status via body mass index (BMI).

### 2.2. Ethical Consideration

The study was approved by the Ethics Committee of the board of directors of University Clinical Hospital (INSALUD-Valladolid, 14 February 2002). The study was carried out following the recommendations of the Declaration of Helsinki, after signing the informed consent by the relatives/guardians of all the patients.

### 2.3. Assessment of Phenotypical Characteristics

We collected the information on age and gender using a nutritional survey. An anthropometric valuation of weight, height, and circumference of the wrist, hip, waist, and mid-arm was performed using standard techniques. Z-scores of weight-for-age, height-for-age, age-for-50° height, weight-for-height, BMI-for-age, BMI-for-height-age, and the mid-arm muscle area, fat-free mass, and fat mass were determined using the references of Frisancho [44], and Orbegozo tables [45]. All four skinfolds (triceps, biceps, subscapular, and suprailiac) were measured using a Holtain Skinfold Caliper. BMI-for-age Z score was used to categorize children as undernourished (<−2 standard deviation: SD), eutrophic (−2 to +2 SD), and obese (>+2 SD). Body composition was assessed by anthropometry and bioelectrical impedance analysis (BIA) (RJL BIA-101(RJL System, Detroit, MI, USA)). We evaluated the bone mineral density (BMD) through bone densitometry by ultrasound (DBM Sonic 1200 IGEA (Emsor S.A., Madrid, Spain)) using the bone conduction speed (BCS) of the last four fingers of the non-dominant hand [46]. To evaluate basal energy expenditure (EE) and resting EE (REE), they were measured by fasting indirect Calorimetry (IC) with a canopy system in standardized conditions (Deltatrac II (Datex-Ohmeda. Helsinki, Finland)). 

### 2.4. Dietary Assessment

The participants gauged and recorded all the food they ingested the week before the blood test. Daily eating of energy; fiber; carbohydrates; protein; lipids; monounsaturated, saturated, and polyunsaturated fats; vitamins A, B1, B2, B6, B12, C, D, E, niacin, and folic acid; and Ca, Mg, iron (Fe), zinc (Zn), and iodine were determined from the food eating records of a 72 h prospective dietary survey (including one of the weekend days). We measured the deficient/adequate nutrient intake by the percentage of Dietary Reference Intake (%DRI) using the Mataix Food and Health software, which allows obtaining the percentage of actual intakes of nutrients following the Spanish recommendations [47,48]. Less than 80% and more than 120% of DRI were the cutoffs utilized to categorize insufficient or elevated dietary intake, respectively. The patients did not receive additional supplementation of Mg or Ca. European Food Safety Authority (EFSA) recommendation of Mg intake for both men and women from 7 to 11 months is 80 mg/day, from 1 to <3 years is 170 mg/day, from 3 to <10 years is 230 mg/day, from 10 to <18 years for males is 300 mg/day and for females is 250 mg/day, and for ≥18 years is 350 mg/day for males and 300 mg/day for females, including pregnant and lactating women [35]. EFSA recommendations (intake reference population, IRP) of Ca intake for both men and women from 7 to 11 months is 280 mg/day, from 1 to 3 years is 450 mg/day, from 4 to 10 years old is 800 mg/day, from 11 to 17 years old 1150 mg/day, from 18 to 24 years old 1000 mg/day, and for adults >25 years old 950 mg/day. From the age of 18, the recommendation includes pregnant and lactating women [49].

### 2.5. Clinical Evaluation

During the clinical examination, we assessed every patient for the presence of symptoms due to altered Mg status. From the first clinical symptoms of Mg deficiency (loss of appetite, nausea and vomiting, fatigue, and weakness) [50] to severe symptoms: neuromuscular symptoms such as muscle weakness, tremors, seizures and paresthesias; cardiovascular abnormalities such as arrhythmia, ventricular fibrillation, and hypertension; and metabolic abnormalities such as hypokalemia and hypocalcemia [51,52]. Patients with weakness, nausea, dizziness, and confusion were also evaluated, ruling them out as mild symptoms of hypermagnesemia. Diminished reflexes, confusion and drowsiness, bladder paralysis, hot flashes, headache, and constipation-like moderate symptoms. In the case of flaccid muscle paralysis, bradypnea, hypotension and bradycardia, PR interval prolongation, AV block, and lethargy are serious symptoms of hypermagnesemia [53,54].

### 2.6. Laboratory Exploration

Fasting venous blood samples were collected to determine the serum level of Ca and Mg by standardized methods. The following cut-off points were used for serum Ca in children (8.8–10.8 mg/dL or 2.2–2.6 mmol/L) [55] and hypercalcemia (>11 mg/dL or 2.7 mmol/L) [56], for Mg: symptomatic hypomagnesemia (<1.22 mg/dL or 0.50 mmol/L), asymptomatic hypomagnesemia (1.22–1.82 mg/dL or 0.50–0.75 mmol/L), chronic latent Mg deficiency (CLMD: 1.82–2.07 mg/dL or 0.75–0.85 mmol/L), interval for health (2.07–2.32 mg/dL or 0.85–0.95 mmol/L), asymptomatic hypermagnesemia (2.32–4.86 mg/dL or 0.95–2.00 mmol/L), symptomatic hypermagnesemia (>4.86 mg/dL or 2.00 mmol/L) [57]. If the serum albumin level was <4.0 g/dL, serum Ca was corrected using the following formula: Ca corrected (mg/dL) = measured Ca (mg/dL) + [4 − albumin (g/dL)] [58]. The cutoff for Ca/Mg intake ratio was 1.70–2.60 [28], serum Ca/Mg ratio was 3.91–4.70 [59], and serum Mg/Ca ratio of 0.4 is optimal (0.36–0.28 too low) [11,60]. Deficiency serum vitamin D < 20 ng/mL, and insufficiency vitamin D 20–30 mg/mL [61]. Serum P in children 4.5–6.5 mg/dL [55]. Hypozincemia levels < 70 µg/dL in children under 10 years of age in both sexes and in females > 10 years and <74 µg/dL in males older than 10 years [62]. Hypocupremia < 70 µg/dL and hypercupremia > 140 µg/dL [63]. The Copper/zinc (Cu/Zn) ratio 0.7 to 1.0 [64]. The Zn/Cu ratio < 4.0 [65].

We evaluated by standard methods: Complete blood count, complete biochemistry, and acute phase protein activity: C-reactive protein (CRP) > 4 U/L and erythrocyte sedimentation rate (ESR) in women > 20 mm/h and men > 15 mm/h. Serum prealbumin ≤ 18 mg/dL, albumin ≤ 3.5 g/dL as visceral protein reserve, transferrin ≤ 200 mg/dL, lymphocytes < 2000 cells/mm^3^, total cholesterol (TC) > 200 (mild–moderate risk) and >225 mg/dL (high risk), and low-density lipoprotein cholesterol (LDL-C) > 115 (mild–moderate risk) and >135 mg/dL (high risk), were considered as cut-off points to evaluate abnormal values. The serum levels of folic acid; beta-carotene; vitamins B12, C, D, E, Ca, P, Fe, Zn [41], and Cu [42]; total immunoglobulin (Ig) G, IgG1–4, IgA, IgM, and IgE; C3 and C4 complement; CD3, CD4, CD8, CD16+56, CD19 lymphocytes, and CD4/CD8 ratio; and insulin-like growth factor-1 (IGF-1) and insulin-like growth factor-binding protein 3 (IGFBP3).

### 2.7. Statistical Analysis

In order to analyze the results, a database was created. The primary variables were Mg and dietary Mg intake. Anthropometric, biochemical (serum Ca, and Ca/Mg ratios), dietary (Ca and Ca/Mg intake ratio), body composition, bone densitometry, and basal EE were secondary variables. Anthropometric (quantitatively and Z-scores) and biochemical results are shown as mean, median, quartiles, SD, and ranges. Two-tailed Student t-test was used to analyze unpaired or paired variables. One-way analysis variance (ANOVA test) and Pearson’s bivariate correlation Test were performed to analyze normally distributed values. We analyzed categorical results by Pearson’s Chi-square test (X^2^) with Yates’s correction and Fisher’s exact test (FET). We used non-parametric tests for variables with non-normal distribution. We calculated odds ratios (OR) to estimate the magnitude of the association between exposure and disease. We performed simple/multiple linear regression analyses to study the significant associations between two or more significant associations. We used the IBM SPSS software version 26.0 (IBM Corp., Armonk, NY, USA) to perform the statistical analysis. The significance level was established at *p* < 0.05 * and <0.01 **.

## 3. Results

Anthropometric, dietary, and biochemical evaluation results from these children and adolescents with chronic illnesses were published previously [41,42]. Table 1 summarizes the baseline demographics and clinic characteristics of the 78 patients (43 females, 55%). The average age was 9.6 ± 4.8 years old (1 to 19 y), 54% of patients (42 participants) were children, and 46% (36 subjects) were adolescents.

Based on nutritional status by BMI, 24, 30, and 24 participants were obese, underfed, and eutrophic, respectively. No one left the study. A total of 99% of these patients came from Valladolid, 96% were Caucasic, 4% were Romani, and 30% of them had polymorbidity (23 cases). The average time of disease was 66 ± 47 months (4 to 182 months), and there were no significant differences in duration of illness between nutritional groups.

Means of serum Ca (9.8 mg/dL or 2.45 mmol/L) and Mg (2.1 mg/dL or 0.86 mmol/L) levels, and dietary Ca (102% DRI) and Mg (105% DRI) intake were regular in the whole series and by group via BMI, without significant differences. All patients had normal serum Ca concentrations. Table 2 shows the number/percentage of patients with deficient/adequate Mg intake versus normal/abnormal serum Mg levels. A total of 45% of patients had hypomagnesemia and 12% hypermagnesemia. A total of 26%cent (20 cases) and 35% (27 subjects) of participants had deficient Mg and Ca intake, respectively. A total of 24% (19 patients) and 28% (22 cases) of patients had high Mg and Ca intake, respectively. Sixteen participants had normal levels of Mg in serum and diet. Therefore, 64% (50 cases) and 79% (62 subjects) of our series had a high risk of Mg deficiency and abnormal Mg status.

The mean serum Ca/Mg ratio (4.79 ± 0.47) was high, and serum Mg/Ca (0.21 ± 0.02) and dietary Ca/Mg intake ratios (1.06 ± 0.51) were low. There was no significant difference between nutritional groups. Fifty-four percent of patients (42 cases) had a high serum Ca/Mg ratio (>4.70). All participants had a low serum Mg/Ca ratio (<0.28). Ninety percent (70 cases) and four percent (3 cases) of this series had a low (<1.70) and high (>2.60) Ca/Mg intake ratio, respectively. Only three participants had normal Ca/Mg ratios (Table 3).

**Figure 2:**
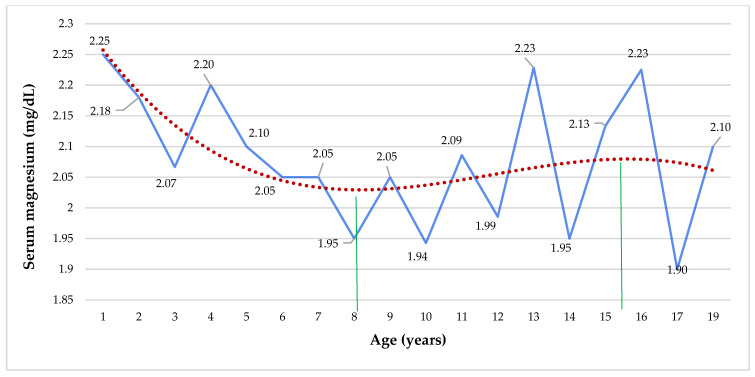
Least Square Means serum magnesium (mg/dL) for factor age (years).

**Figure 3:**
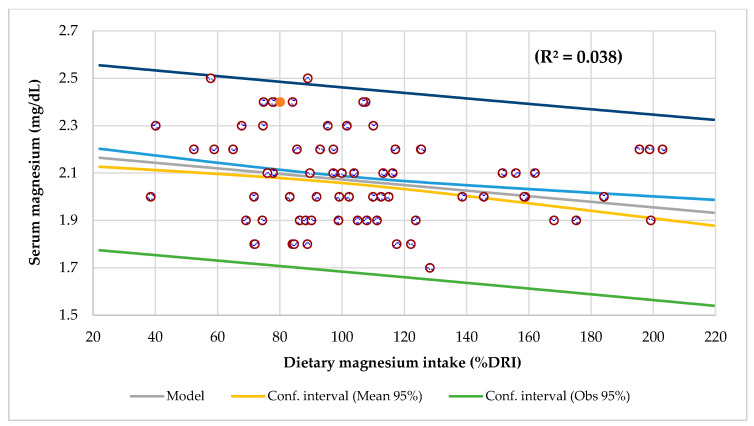
Regression of serum magnesium level (mg/dL) by dietary magnesium intake (%Dietary Reference Intake).

The mean serum Mg by age presents a polynomial curve, with a tendency to decrease from 1 to 8 years, and then there was an increase slightly until 15 and 16 years, where it tends to decline again (Figure 2). Dietary intake and serum Ca levels were inversely and significantly associated with age (Figures S1 and S2). Serum Mg level had a negative and significant association with Mg intake when adjusted for age (R^2^ = 0.038, *p* < 0.0001) (Figure 3), and there was a trend for dietary Mg intake to decrease with age (R^2^ = 0.050, *p* = 0.050). 

Table 4 shows the most significant characteristic among participants with hypermagnesemia both infants were malnourished, one had inadequate Ca and Zn intake, and the other had deficient iodine intake. Another underweight 12-year-old girl had stunted growth, and low Mg, Zn, and iodine intake. One eutrophic 2-year-old boy had inadequate Zn and iodine intake, high LDL-cholesterol, ESR, hypercupremia, and a high Cu/Zn ratio in ranges of severe inflammation. Another eutrophic 13-year-old girl had poor Ca, Mg, zinc, and iodine intake, and high cholesterol and LDL-cholesterol. One obese 16-year-old girl had insufficient Ca, Mg, and Zn intake and high LDL cholesterol. The last three subjects were at high CV risk. One obese 13-year-old boy had limited calcium, Mg, Zn, and iodine intake. Two obese 13-year-old patients (boy and girl) had inadequate Ca and iodine intake, high ESR, and deficient nutritional zinc status (low serum and consumption). The boy had leukopenia, and lymphopenia.

Table 5 displays the differences among subjects with chronic sicknesses in the entire series. The mean serum Ca (*p* = 0.021) and Mg levels (*p* = 0.036) and dietary Ca intake (*p* = 0.044) were significantly higher in children under 5 years old and in patients with an elevated ESR for serum Mg (*p* = 0.024) and Ca (*p* = 0.045) than in participants ≥ 5 years old and subjects with normal ESR. The mean serum Ca levels (*p =* 0.015), dietary Ca (*p* = 0.000), and Mg (*p* = 0.000) intake were significantly higher in children than in adolescents. Hypermagnesemia patients had significantly lower Mg intake than hypomagnesemia (*p* = 0.001) and normal serum Mg (*p* = 0.032) participants. There was a significant difference in mean serum Mg and serum Ca/Mg ratio as a function of serum Mg levels. Serum Mg and its intake, diet Ca intake, and Ca/Mg ratios were significantly different according to dietary Mg intake levels.

Regarding the intake of the dietary survey, the diet for the entire series was high protein (276% DRI), high cholesterol intake (266% DRI), slightly low carbs intake (79.5% DRI), normal total lipids (111% DRI), with a deficient consumption of Zn and iodine. Table 6 displays the OR in the whole series. The likelihood of finding dietary Mg deficiency was higher in subjects with microcephaly, children, patients with inadequate energy, total fat and calcium intake, and higher consumption of vitamin B1, B2, niacin, and iron than in subjects with regular Mg intake. The probability of finding high dietary Mg intake was overhead in participants with lower vitamin E, Zn, and iodine consumption and higher calcium intake than in patients with regular Mg intake.

Table 7 displays the association between BCS by BIA with anthropometric assessment. The mean BCS (1924 ± 88) was standard in all series, and there were no significant differences in the groups by BMI. In the whole series, only five subjects had low BMD by BCS, and thus a high risk of osteoporosis. Regression analysis showed that BCS in our series had a significant relationship with dietary Ca (*p* = 0.017) and Mg intake (*p* = 0.048). By groups, BCS was associated with serum Ca (*p* = 0.032), serum Ca/Mg ratio (*p* = 0.049), and Mg intake (*p* = 0.004) only in the obese group. In the entire series, BCS had a significant association with age, body weight, and height, and kg of muscle and fat mass by anthropometry. These were not the case with BMI and weight-for-height associated with BCS only in the eutrophic group. Fat and muscular mass by BIA had a meaningful association with BCS in the obese and eutrophic groups [42].

Table 8 and Tables S1–S5 (Supplementary Materials) show the linear and multilinear regression analysis between serum and dietary levels of Ca and Mg and Ca/Mg ratios in the whole series, by the nutritional group and age group (children and adolescents).

## 4. Discussion

Surprisingly, there are few studies related to Mg status in chronic illnesses in childhood and adolescence. As far as we know, this is the first study to explore serum and diet Mg levels and their association with nutritional biomarkers in a series of children and adolescents with chronic illnesses. The results displayed that the mean serum Ca and Mg levels and their dietary intake were normal in the entire series and by nutritional groups. Forty-five percent of participants had hypomagnesemia, and twelve percent had hypermagnesemia. No patients had abnormal serum Ca levels. A total of 35%and 26% of subjects had deficient Ca and Mg intake, and 28% and 24% of patients had elevated Ca and Mg intake, respectively. No participants with high Mg intake had hypermagnesemia. Only five patients had serum Mg deficiency and poor Mg intake, and sixteen subjects had normal levels of Mg in serum and diet. Serum Mg level had a significant inverse relationship with diet Mg intake when adjusted for age, and there was a trend for dietary Mg intake to decrease with age. Dietary intake and serum Ca levels were inversely and significantly associated with age. The mean serum Ca/Mg ratio (4.79) was high, and the mean dietary Ca/Mg intake ratio (1.06) was low. Fifty-four percent and ninety percent of our series had an elevated serum Ca/Mg ratio (>4.70) and a low Ca/Mg intake ratio (<1.70), respectively. Therefore, in this study, 64% of children and adolescents with chronic conditions had a high risk of deficiency Mg status (low levels in serum and diet). Seventy-nine percent of them would be at elevated risk of a state of abnormal Mg and developing other chronic diseases and cancer (anomalous Ca/Mg ratios).

It is necessary to consider that only a few studies on children and adolescents with chronic sicknesses exist. Although it is difficult to estimate, various studies state that for children aged less than 18 years, about 16% have poor oral health, from 7% to 10% have asthma, 4% have food allergies, 0.7% have seizure disorders, and 0.3% have DM [66]. We performed a study on 78 patients (9.6 ± 4.8 years, 46% adolescents) with chronic illnesses (30% with polymorbidity), 99% were from Valladolid, 96% were Caucasian, and 4% were Romani. Thirty-one percent of them (24 cases) were obese and eutrophic, and thirty-eight percent (30 subjects) were underfed. The prevalence of diseases was malnutrition, syndromic diseases, encephalopathies, KD, hyperlipidemia, IDDM, and eating disorders. There were no meaningful differences in the duration of the disease (mean 66 months) between the nutritional groups. These results agree with a study performed on 2961 patients under 18 years of age (9.5 ± 4.7 years) in an elemental health area of the Madrid Primary Care, where 15.7% of subjects (423 cases) were chronic. In contrast, 11.3% had multiple morbidities, and the most prevalent illnesses were asthma (6.1%), attention deficit hyperactivity disorder (ADHD) (1.8%), and obesity (1.4%) [67].

### 4.1. Serum Magnesium Levels

Studies show that the critical Mg range is below <1.0 mg/dL (or 0.5 mmol/dL) and >4.9 mg/dL (or 2.0 mmol/L) [53]. In adults, a reference range of 1.82 to 2.31 mg/dL (0.75 to 0.95 mmol/L) is common [57]. In children, Pagana et al., recommended limits of 1.70 to 2.06 mg/dL (0.69 to 0.85 mmol/L) for serum Mg [55]. For the prevention of Mg deficiency, the German Society for Mg Research e.V. proposed a lower limit value of 1.94 mg/dL (0.80 mmol/L) [68]. Our findings showed that the mean serum Mg (2.10 mg/dL or 0.86 mmol/L) and Ca (9.90 mg/dL or 2.47 mmol/L) levels were normal and there were no significant differences by gender and nutritional groups. The mean serum Mg in our adolescent group (2.08 ± 0.22 mg/dL or 0.85 ± 0.09 mmol/L) had a significant difference with the mean serum Mg (1.92 ± 1.01mg/dL, or 0.79 ± 0.42 mmol/L, *p* = 0.000) of 2447 Mexican adolescents (12 to 19 years), who also had no differences by gender. Epidemiological statistics point out that the risk of several diseases rises with decreasing serum Mg, even within the reference range of 1.82 to 2.31 mg/dL (0.75 to 0.95 mmol/L) [41]. Current data suggest an increased risk of CVD, T2D, and mortality rate from these NCDs even at values below 1.82 to 2.07 mg/dL (0.75 to 0.85 mmol/L) [57]. Internationally and supported by metabolic and equilibrium studies, a lower limit value of 2.07 mg/dL (0.85 mmol/L) is postulated [42], according to which there would be a Mg deficiency in a range of >1.82 to 2.07 mg/dL (0.75 to 0.85 mmol/L) [41,69].

Consequently, depending on the cut-off point used, the incidence of patients with hypo or hyper Ca and Mg varies. In our study, no patient had hypo or hypercalcemia. One per cent of participants presented hypomagnesemia < 1.70 mg/dL (0.70 mmol/L) [70], a percentage similar the reported incidence of 2% in the general population [71]. Conversely, if we use the cut-off point ≤ 1.82 mg/dL (0.75 mmol/L) suggested by several studies [57,68,69], the incidence of cases of hypomagnesemia rises to 10% (eight subjects). This result is comparable to the 8.8% risk of hypomagnesemia found in 3421 subjects between 24 and 60 years old from Andalusia, Spain [72]. Furthermore, if we adopted the evidenced-based reference interval for serum Mg < 2.07 mg/dL (0.85 mmol/L) to reduce the risk of CVD, T2D, and other chronic diseases [57,69], 45% of our series (47% of teenagers) had serum Mg deficiency (35 cases). That is, 10% (eight patients) had asymptomatic hypomagnesemia from 1.22 to 1.82 mg/dL (or 0.50–0.75 mmol/L), and 35% (27 subjects) had CLMD from 1.82 to 2.07 mg/dL (0.75–0.85 mmol/L) [57,73]. This result differs from the 37.6% overall prevalence of serum Mg deficiency in 2447 Mexican adolescents [74]. As in our study, in 20,438 hospitalized adult patients, serum Mg deficiency was found in 7%, 25%, and 60% of them, utilizing cut-off values of 1.58, 1.82, and 2.07 mg/dL (0.65, 0.75, and 0.85 mmol/L), respectively [75].

We must take into consideration that Mg deficiency may be previously present in a very advanced state when the diagnosis of hypomagnesemia is made [76]. It can cause neuromuscular, cardiac, or nervous disorders, resulting in neuromuscular dysfunction, symptoms of muscle weakness, and muscle cramps [77]. Mg deficiency is quite common; although, symptoms only appear when the critical value of 1.0 mg/dL (0.5 mmol/L) is exceeded [53,78]. This circumstance may be the reason why no child or adolescent in our series presented symptoms related to hypomagnesemia [50,51,52]. A hypomagnesemia deficient state depends mainly on alterations in intake, redistribution, and excretion [52] of Mg, in addition to pre-existing pathological conditions. It is common in subjects with impaired gastrointestinal (GI) absorption, for example, in celiac disease [79] or CF [43,80], inflammatory bowel diseases [81], or the presence of colon cancer, gastric bypass, and other minor GI disorders [82]. Other causes of Mg deficiency include IDDM, KD, fluid and electrolyte imbalances [83], migraine, osteoporosis, asthma, pre-eclampsia, and CVD [84,85]. It is important to note that substantial fluctuations in serum Mg concentrations are associated with an increased mortality rate (*p* = 0.017) [75]. Therefore, 45% of children and teenagers of this series may have an increased risk of morbidity and mortality due to CVD [13,18,19], T2D [21], neurodegenerative diseases [22], other chronic diseases [15,16,17,20,22,23,24], and even cancer [15,22,25]. Mg deficiency is related to a few chronic sicknesses, such as headache, cardiac arrhythmias, HTN, sleep disturbances, depression, pregnancy complications, IR, abnormal glucose tolerance, vitamin D deficit, and secondary electrolyte disorders (hypocalcemia, hypokalemia) [84]. Only when the Mg stores are depleted, as in the case of advanced deficiency, will serum Mg decline [86].

It is interesting to find that according to the cut-off point used, the upper limit of serum Mg would be 2.43 mg/dL (1.00 mmol/L) [86] or 2.67 mg/dL (1.10 mmol/L) [12,87]. Whether we used the cut-off suggested by Pagana et al. of >2.07 mg/dL (0.85 mmol/L) [55], 50% of our series of children and adolescents with chronic diseases had hypermagnesemia. Despite this reality, the upper limit used was 2.32 mg/dL (0.95 mmol/L) [57], obtaining a not-insignificant 11% of subjects (9 cases) with hypermagnesemia. In contrast, in 101 HD patients with CKD, the mean serum Mg was 3.21 mg/mL (1.32 mmol/L), and hypermagnesemia (>3.65 mg/dL or 1.5 mmol/L) cases was found in 17 (16.8%) patients [88]. In our series, mean serum Mg was significantly higher in patients with hypermagnesemia and standard levels than in those with hypomagnesemia. Hypermagnesemia is a rare but severe electrolyte disorder [53] with neurologic and cardiac abnormalities [54]. Hypermagnesemia shows no symptoms up to a serum Mg of 4.86 mg/dL (2.00 mmol/L) [13]. According to Cascella and Vagar, patients with mild hypermagnesemia, asymptomatic or paucisymptomatic happen with values < 7 mg/dL (2.88 mmol/L), moderate between 7 and 12 mg/dL (2.88–4.94 mmol/L), severe hypermagnesemia > 12 mg/dL (4.94 mmol/L), and values > 15 mg/dL (6.17 mmol/L) cause coma and cardiorespiratory arrest [53].

Hypermagnesemia usually occurs in patients with severe renal failure or with excessive Mg intake, for instance, in the form of Mg-containing antacids or laxatives [89]. In our series, 33% of hypermagnesemia patients were undernutrition, and 22% were eutrophics; 67% were females, adolescents, and had deficient Ca consumption; 45% were obese, and 45% had inadequate Mg intake; and 89% had deficient Zn, and 89% had deficient iodine intake. It was curious that the eutrophic 2-year-old boy had poor Zn and iodine intake, high LDL-cholesterol, ESR, hypercupremia, and a high Cu/Zn ratio in ranges of severe inflammation. Two obese 13-year-old patients (boy and girl) had inadequate Ca and iodine intake, elevated ESR, and deficient nutritional Zn status (low serum and consumption). The boy had leukopenia and lymphopenia. One obese 16-year-old girl had insufficient Ca, Mg, and Zn intake and high LDL cholesterol. Patients with hypermagnesemia can present neurologic and cardiac abnormalities, including confusion, coma, widening of QRS complex, PR interval prolongation, and cardiac arrest [54]. Despite the fact that 11% of children and adolescents in this series had mild hypermagnesemia, no one had any symptoms.

Total plasma Mg concentrations are remarkably constant in healthy subjects throughout life, while whole body Mg and Mg in the intracellular compartment tend to decrease with age [14]. Although in our series, there were no substantial differences in the mean serum Ca and Mg by gender, serum Mg (*p* = 0.036) and Ca (*p* = 0.021) levels were significantly higher in children under 5 years old than in patients ≥ 5 years old. Serum Ca levels were meaningful higher in children than in adolescents (*p* = 0.015) and decrease with the age (Figure S1). Lietman et al. pointed out that serum Ca levels are bigger in children than in adults [90]. Moreover, serum Mg by age displayed a polynomial curve, with a trend to decrease from year 1 to 8 years and then a rise slightly until 15 and 16 years, where it tends to decline again (Figure 2). Quite the reverse, in a group of CF patients, serum Mg levels reduced significantly with age (R^2^ = 0.234) [43]. According to Malinowska et al., in a series of 20,438 hospitalized patients, it was observed that serum Mg levels did not depend on gender and that there was a slight positive relationship with age (*p* < 0.0001, *r* = 0.07) [75]. Moreover, we did not find significant differences between serum Mg levels by nutritional status. On the contrary, in a series of 140 children (2–14 years), the serum Mg concentration was meaningfully lower in the overweight and obese group (2.08 mg/dL or 0.85 mmol/dL) as compared to the normal weight group (2.55 mg/dL or 1.05 mmol/dL, *p* < 0.001) [91]. If we compare our results with that series of children, we do not see significant differences between the two groups of children with overweight/obesity (*p* = 0.709). However, the serum Mg level of the normal-weight children group was meaningfully higher than those of our three groups (*p* = 0.000) [91], showing that our series of young population with chronic illnesses have lower levels of serum Mg.

### 4.2. Phenotypical Characteristics

In the whole series and the obese group, serum Mg and Ca/Mg intake ratio had no association with phenotypical characteristics. However, head and mid-arm circumference had a significant association with serum Ca and Ca/Mg ratio. Height-for-age was associated with Ca and Mg intake. Furthermore, fat mass and kg mass muscular by BIA were correlated with serum Ca and Mg intake, respectively. By groups, we need to highlight the significant association of height-for-age, weight-for-age, weight-for-height, BMI, waist (ratios) and head circumferences, muscular mass by anthropometry with Mg; BMI, subscapular skinfold, head and hip circumference, fat mass by BIA and fat/muscular mass ratio with Ca; and height-for-age, weight-for-age, BMI, waist (ratios), and head circumferences with Ca/Mg ratios. In addition, diet Mg intake had associations with the sum of the skinfold Z-score and waist/hip ratio (R^2^ = 0.438) and bicipital skinfold and waist/hip ratio (R^2^ = 0.471). Diet Ca intake with height-for-age and bicipital skinfold (R^2^ = 0.391), the sum of skinfolds, Z-score of BMI, and height-for-age and head circumference (R^2^ = 0.705). Only in the undernourished group serum Ca/Mg ratio was associated with waist/height and waist circumference (WC, R^2^ = 0.374), and Ca/Mg intake ratio with Z-score of heigh-for-age, BMI, and suprailiac skinfold (R^2^ = 0.736). It is interesting to note that the likelihood of finding dietary Mg deficiency was higher in subjects with microcephaly (OR 3.6). By age group, waist/hip ratio was significantly associated with Mg status in children and with dietary Ca in adolescents.

Usual Ca consumption displayed a weak significant positive relation with the height Z-score in the total sample (*r* = 0.126; *p* = 0.000). Cuadrado-Soto et al. reported that this direct association occurred in children aged 4 to 5 years (*r* = 0.105; *p* = 0.018) and those 6 years and older (*r* = 0.134; *p* = 0.003) [92]. According to Cormick et al., Ca fortification of foods increased height in children (moderate-certainty evidence). This effect may have a potentially crucial public health impact on growth retardation and the prevention of bone disease [93]. BMI also may affect Mg status, particularly in children and women [86]. Farhangi et al. showed that Mg deficiency in menopausal women was associated with an increased BMI [94]. In 210 T2D patients aged 65 years and above, Mg intake was inversely correlated with (WC), body fat percent, and BMI (*p* < 0.005) [95]. According to Rafiee et al., insufficient intake of Mg is associated with an augmented waist/hip ratio. A meta-analysis of twenty-eight randomized controlled trials (RCTs) studying the impact of Mg supplementation on anthropometric assessments in adults showed a reduction in WC assessed in those who were obese (BMI > 30 kg/m^2^) [96]. Furthermore, Hassan et al. found a significantly strong inverse relationship between serum Mg levels and BMI [91]. These facts support the idea, as in this study, of continuing to evaluate anthropometric markers in patients with abnormal Mg status to understand their relationship.

### 4.3. Bone Densitometry

Ca, P, Mg, and vitamin D play essential roles in bone mass growth and development, especially in the pediatric stage [97]. Although serum levels (Figure S1) and dietary Ca (Figure S2) intake in the whole series were inversely and significantly associated with age; in other studies, Ca intake increased with age [98], such as in the EsNuPI study (*r* = 0.283) [92]. Especially in women, the steady decline in Ca levels with age probably reflects the gradual age-dependent decline in its absorption, especially in postmenopausal women when estrogen production declines [99]. Dietary Ca intake in low-income settings is generally low, and probably about 3.5 billion people are at risk of Ca deficiency [100]. Ca shows a crucial role in skeleton maintenance, with the body’s Ca 99% found in the form of hydroxyapatite [101]. In bone, Mg exists as a hydroxyapatite crystal structure, which plays a crucial role in keeping normal serum Mg concentrations and can become affected at the time of Mg deficiency [102]. Furthermore, the bone Mg content decreases with age, and only a third of the Mg is available for ion exchange to maintain the extracellular level [50,84].

In this series, BCS had a significant relationship with age, body weight, and height, and muscular and fat mass by anthropometry. Nevertheless, this was not the case with BMI and weight-for-height associated with BCS only in the eutrophic group. Fat and muscular mass by BIA had a meaningful association with BCS in the obese and eutrophic groups [42]. Results show that the mean BCS of 1924 was normal in all series and by groups, age, and gender. Only five cases had low BCS, and thus a high risk of osteoporosis (Table S4). During bone growth and development, adequate intake of the nutrients listed above can help achieve the optimal peak of bone mass, which helps prevent the development of osteoporosis [103]. Serum levels and dietary Mg intake in this series had no significant differences between patients with low/normal BCS. In the entire series, BCS had a meaningful association with serum Ca (R^2^ = 0.081) and Mg intake (R^2^ = 0.057). BCS had associated with Mg intake (R^2^ = 0.368), serum Ca (R^2^ = 0.220), and serum Ca/Mg ratio (R^2^ = 0.190), only in the obese group. Orchard et al. reported a positive relationship between diet Mg intake and BMD [104]. In 63 healthy children (4 to 8 years), diet Mg intake but not Ca intake had a significant correlation with both total bone mineral content and density [105].

The most important deposits of Mg are the bones (60%), the muscle (20%), and the soft tissues (19%) [106]. During growth, the body requires an intake of 6 mg/kg body weight per day for Mg retention of 3 mg/kg body weight per day [99]. In our study, IGF-1 had a meaningful association with dietary Mg intake in the entire series (R^2^ = 0.039), and the obese group (R^2^ = 0.309), and IGFBP3 had a significant association with the Ca/Mg intake ratio in the obese group (R^2^ = 0.270). Proteins and micronutrients such as Zn, potassium (K), Mg, and vitamin D influence growth hormone (GH) and IGF-1, both components are involved in critical points in growth periods and bone development [107]. Subclinical or chronic Mg deficiency is often underestimated because serum Mg does not reflect reduced levels of Mg within cells and bone, but rather extracellular Mg [108].

Few studies informed the association between Mg intake and growth that would support our findings. Guatemalan children with edematous protein-caloric undernutrition had a bigger recovery rate after Mg supply [109]. Singla et al. informed that undernutrition children had deficient Mg levels concerning their non-malnourished counterparts [110]. In animal models, Mg deficiency had associated with low plasma IGF-1, which returns to normal levels once the deficiency is corrected [111]. Vitamin D metabolites may be affected (secretion and/or responsiveness) in the face of a severe state of Mg deficiency [112]. Moreover, in rats, the formation and function of vitamin D were altered with the reduction in Mg intake [113]. Mg deficiency can have a deleterious effect on bone health, directly by increasing osteoclast and decreasing osteoblast activity and indirectly interfering with vitamin D and parathyroid hormone (PTH). These facts favor inflammation and the consequent bone loss [114].

### 4.4. Dietary Intake Survey

An inadequate intake of Mg is the most likely culprit that leads to CLMD [115,116]. Mg homeostasis is tightly controlled and depends on the dynamic balance between intestinal absorption, kidney excretion, and bone storage [117]. Mg deficiency is mainly due to a reduction in the intake, impaired absorption, and/or a bigger excretion from the body [70]. During pregnancy, there is an increased health risk to both the mother and the newborn, fetal and intrauterine growth restriction, gestational diabetes, preterm labor, preeclampsia [118], and MetS [119], with implications that may extend to the offspring’s adulthood. In this study, serum Mg level had a meaningful inverse association with diet Mg consumption when adjusted for age (R^2^ = 0.038) (Figure 3). In 101 CKD patients in hemodialysis (HD), the daily intake of Mg was higher by 31.7% in the group with serum Mg >3.65 mg/dL or 1.50 mmol/dL. There was a strong direct association between Mg intake and serum concentration in the whole group (*r* = 0.870). In these patients, Mg consumption was the most important determinant of serum Mg levels [88].

In contrast, in 62 individuals, the daily Mg intake correlated with serum Mg concentration [120], and in 27 strictly controlled metabolic studies, age did not affect Mg absorption [121]. Moreover, in 68 adults with HTN, no correlations between serum Mg and dietary Mg intake levels were found [122]. By age groups, children presented a significant association between serum and dietary levels of Ca and Mg and Ca/Mg ratios, mainly with vitamins A, B1, and E and Ca, and adolescents with the intake of Mg and Ca. Dietary Mg intake and albumin levels can influence serum changes and short-term changes, such as variability in the amount of Mg absorbed and excreted [123]. In addition, whereas intestinal absorption of Mg tends to decrease with age, urinary excretion of Mg tends to increase [124]. Older adults have lower dietary intakes of Mg than younger adults [125]. These circumstances could partly explain our results and the fact that these children and adolescents with chronic conditions may be deficient in Mg long before the beginning of their chronic disease.

Additionally, results showed that the average diet in our series was high in protein, and cholesterol, slightly low in carbs, with a deficient intake of Zn and iodine. Although dietary Ca (102% DRI) and Mg (105% DRI) intake were regular, and there was no significant difference by nutritional group and gender, 26% (20 cases) and 35% (27 subjects) of participants had deficient Mg and Ca intake, respectively. There was a trend for dietary Ca (R^2^ = 0.089) and Mg (R^2^ = 0.050) to decline with age, and children had a dietary Ca (*p* = 0.015) and Mg (*p* = 0.000) intake meaningfully higher than adolescents and the likelihood of finding dietary Mg deficiency was higher in children < 10-year-old (OR 1.4) than in subjects with regular Mg intake. Diet Ca intake was higher in children < 5 years (*p* = 0.044) than in participants > 5 years. These results are interesting and are not consistent with findings found in a study conducted in Spain. The Anthropometry, Intake, and Energy Balance in Spain (ANIBES) study discovered that the mean Ca and Mg intake in the inhabitants (aged 9–75 years) was low, indicating that 76% and 79% of the population were eating <80% of the national RDA [126]. In addition, in 1448 Spanish children (1 to <10 years) in the EsNuPI (Estudio Nutricional en Población Infantil Española) study, boys (194 mg/day) had a meaningfully bigger intake than girls (188 mg/day), and Ca (*r* = 0.283) and Mg (*r* = 0.316) eating augmented with age (*p* < 0.001) [92]. The patients’ diet for their chronic conditions may be the reason for our results. 

We need to consider that only 21% (16 cases) of our patients had normal serum levels and diet Mg intake, and 6% (5 subjects) had deficient serum levels and dietary Mg intake. In addition, 24% (19 subjects) and 28% (22 cases) of patients had high Mg and Ca consumption. Patients with an elevated dietary Mg intake had a significantly lower serum Mg level than patients with a normal and deficient Mg intake (*p* = 0.018), and participants with normal and abnormal Mg intake had significantly different mean diet Mg intake levels. Out of 50% of the patients who presented an adequate Mg consumption (39 cases), fourteen had a serum Mg deficiency (18%) and four had hypermagnesemia (5%). Of the other 26% of the subjects with deficient Mg intake (20 cases), five had serum Mg deficiency (6%), and four had hypermagnesemia (5%). Hypermagnesemia patients had significantly lower Mg intake than hypomagnesemia (*p* = 0.001) and normal serum Mg (*p* = 0.032) ones. Interestingly, despite 24% of patients having a high Mg intake (19 cases), no one had hypermagnesemia, and only twelve had serum Mg deficiency (15%). One possible reason for patients with high Mg intake without hypermagnesemia is that the kidney has a large capacity for Mg clearance. Hypermagnesemia usually occurs in patients with renal failure and excessive Mg intake [89].

It is interesting to point out that the likelihood of finding dietary Mg deficiency was higher in subjects with microcephaly (OR 3.6), patients with inadequate energy (OR 4.2), total fat (OR 4), and Ca (OR 4.2) consumption, and higher consumption of vitamin B1 (OR 5.8), B2 (OR 3.7), niacin (OR 7.9), and Fe (OR 3.2) intake than in subjects with regular Mg intake. Alternatively, the probability of finding high dietary Mg intake was overhead in those with lower vitamin E (OR 3.3), Zn (OR 6.6), and iodine (OR 4.3) consumption and higher calcium (OR 4.3) intake than regular Mg eating. The uptake of Mg can be influenced by physiological factors, such as age and the other food components in a meal, including proteins, medium chain triglyceride (MCT), and low- or indigestible carbs such as resistant starch, oligosaccharides, inulin, mannitol, and lactulose enhance Mg uptake [127]. Higher protein intake increased Mg absorption, possibly by preventing the precipitation of Ca–Mg–P complexes in the ileum, resulting in an increase [128]. However, inhibitory effects can be exerted by high levels of partly fermentable fibers (i.e., hemicellulose), non-fermentable fibers (i.e., cellulose and lignin), and phytate and oxalate [127].

Mg is a crucial divalent metal ion for living organisms [84] and it is required as a cofactor for more than 600 enzyme reactions involved in fat, protein, and carb metabolism and supports the action of insulin [129]. Regarding the nutritional survey, only vitamin E intake in the entire series had a meaningful association with serum Mg levels. Supplementation of Mg plus vitamin E also significantly decreased serum triglycerides and VLDL-cholesterol levels [130]. Furthermore, diet Mg intake had an association with energy, vitamin B12, folic acid, Zn, vitamin E, and cholesterol intake (R^2^ = 0.650), diet Ca intake with calories, proteins (R^2^ = 0.274), and Fe intake, and Ca/Mg intake ratio with Mg, niacin, Fe, and protein intake (R^2^ = 0.391). By groups, we highlight that serum Mg was associated with polyunsaturated fat intake in the obese group, and cholesterol, carbs, and vitamin B1 intake (R^2^ = 0.407) in the eutrophic group. The intake of Mg was associated with the consumption of Ca, monounsaturated fats, proteins, folic acid, and cholesterol (R^2^ = 0.824, obese group), with the intake of vitamin B12 and Zn (R^2^ = 0.759, malnourished group), and with the energy and iodine intake (R^2^ = 0.696, eutrophic group). Diet Ca intake with Fe (underfed group) and saturated fat intake (eutrophic group). Serum Ca/Mg ratio with monosaturated fat intake (underfed group), and cholesterol and carbs intake (R^2^ = 0.477, eutrophic group). Diet Ca/Mg intake ratio with Mg and vitamin B2 intake (R^2^ = 0.445, obese group), Mg and Fe intake (R^2^ = 0.529, underfed group).

In 210 T2D patients aged 65 years and above, Mg intake had a positive association with energy (*r* = 0.520) and protein intake (*r* = 0.646), and inversely correlated with triglyceride [95]. Nonetheless, although the overall median Mg intake adequacy in 2447 Mexican adolescents was 85%, no significant associations were found with gender, BMI, or CRP [74]. A meta-analysis and systematic review indicated that dietary Mg intake is inversely associated with serum CRP levels [131], and saturated fat and niacin intake (R^2^ = 0.664, eutrophic group). Energy intake was significantly correlated with the intakes of Ca (*r* = 0.48) and Mg (*r* = 0.74), and protein consumption with plasma Ca levels (*r* = 0.13) [132]. In a cross-sectional survey carried out in Andalusia, in a random sample of 3421 subjects, between 24 and 60 years, energy intake was significantly correlated with Ca (*r* = 0.48, *p* < 0.01), and Mg (*r* = 0.74, *p* < 0.01) intake. They reported a meaningful association between protein intake and plasma Ca (*r* = 0.13, *p* < 0.05) levels [72]. Mg supplementation combined with vitamin E, which effectively scavenges the peroxyl radical in cell membranes to inhibit lipid peroxidation, has been hypothesized to exert a synergistic effect on glycemic control [133]. Lipids impact the absorbability of Mg, whereby the lipid composition is suggested to be the influencing factor [134]. Specifically, the regular consumption of dairy products and milk formulas are foods of high nutritional value that provide high amounts of Ca, Mg, P, and vitamin D, which are chief factors in bone health [135].

### 4.5. Biochemical Analysis

Hereinafter, the results shown in Table 8 and Tables S1–S3, on the significant association between Ca and Mg levels in serum and diet and Ca/Mg ratios with biochemical indicators in the entire series, by nutritional group and age group, will be analyzed. In addition to the intestine, bone, and kidney, vitamin D, PTH, and estrogen are involved in Mg metabolism [106]. Hypomagnesemia significantly elevates the levels of alkaline P and PTH in patients with CKD stage 5 under maintenance HD [136]. Mg is necessary for lecithin cholesterol acyltransferase and lipoprotein lipase functions, which reduce triglyceride levels and increase HDL-cholesterol levels. Mg-ATP is also the limiting enzyme control factor in cholesterol biosynthesis [137]. In elderly people, Mg consumption was positively correlated with HDL (*r* = 0.192). Mg intake had significant inverse relationships with triglyceride (*r* = −0.144) and WC (*r* = −0.243) [95]. In 1318 healthy adult subjects recruited from the Newfoundland population, serum Mg was positively correlated with age, and serum P, Ca, albumin, total cholesterol, HDL-cholesterol, LDL-cholesterol, and triglyceride levels [138]. In a case-cohort study of 4443 (aged 40–75 years) of the EPIC (European Prospective Investigation into Cancer)-Norfolk cohort, a significant inverse association with TC was observed. A higher Mg intake has been related to beneficial increases in HDL-cholesterol concentrations [139]. Early studies reported that increasing Ca in the diet significantly depressed Mg absorption [140]. The same depressive effect on Mg absorption was shown with excess P, Fe, Cu, Mn [141], and Zn [142]. Folic acid or folate (natural form) is an essential micronutrient for many physiological processes. Serum folate level (≥33.0 ng/mL) and a lower Ca/Mg ratio were associated with worse assisted reproductive technology (ART) outcomes in normogonadotropic women. A baseline lower Ca/Mg ratio (<4.55) was associated with worse clinical ART outcomes in a dose-dependent manner [143].

### 4.6. Blood Analysis and Inflammatory Response

In connection with the blood account, the mean CRP and ESR levels were normal. Eight participants (11%) had CRP levels. A total of 24% of patients had high ESR (19 cases), 16% of them had hypermagnesemia (3 cases), 26% had deficient Mg intake (5 patients), and 21% had high Mg consumption (4 subjects). The Ca (*p* = 0.045) and Mg (*p* = 0.024) level were meaningfully higher in patients with an elevated ESR (*p* = 0.024) than in participants with normal ESR. In 75 patients with the Kellgren–Lawrence Grade 1–4 knee OA, serum Mg level is not correlated with the inflammatory markers including ESR and CRP in this patient population [144]. The elements Mg, selenium (Se), Zn, Mn, and Cu are involved in the mechanisms of cellular antioxidant defense [145]. Moderate or marginal (subclinical) deficient Mg intake generally is asymptomatic. Animal studies, in contrast, indicate that a deficient Mg intake primes phagocytic cells for the release of proinflammatory cytokines leading to chronic inflammatory and oxidative stress [146].

Mg is closely related to the immune system in both nonspecific and specific immune responses (i.e., innate and acquired immune responses) [147]. Mg acts in the acute phase response and the function of the macrophage response to cytokines [148]. It influences the development, differentiation, and proliferation of lymphocytes [149]. In an Mg-deficient diet, absorption switches to active transcellular transport in the large intestine mediated by Transient Melastatin Receptor Potential Channel 6 and 7 (TRPM 6 and 7) [52]. This Mg transporter TRPM7 seems to play a distinct role in cells’ development. In cells lacking this transporter, and thus the Mg supply, developmental inhibition, and early cell death occurred [150]. Erythrocytes, PLT, and Hb are significantly and substantially higher in the presence of higher serum Mg in patients without T2D and without central obesity [151]. 

It is important to note that Mg is vital in acquired immunity by regulating lymphocyte growth [147], and function [152]. Low levels of CD4+ T-lymphocytes and their defective activation are due to the decreased Mg influx, which does not activate PLCγ [153]. This component acts as a cofactor for immunoglobulin synthesis, CI 3 convertase, antibody-dependent cytolysis, macrophage responses to lymphocyte, IgM lymphocyte binding, T helper B cell adherence, substance P binding with lymphoblast, and uptake binding to macrophage [154]. Mg deficiency results in a stress condition that activates the sympathetic system and the hypothalamic–pituitary axis, causing fat accumulation and release of neuropeptides. These results in the immune response followed by inflammatory cascades [155]. Hypomagnesemia contributes to oxidative stress and inflammation and is inversely related to CRP [156]. An average adult human body contains about 24 g or 1000 mmol of Mg, of which 99% is stored, and 1% is available extracellularly in serum and red blood cells (RBC) [50]. In the case of Mg deficiency, a normal serum level is sustained by extracting it from the RBCs. Thus, RBC Mg is considered to be a biomarker for Mg deficiency [70].

### 4.7. Calcium/Magnesium Ratios

Serum Mg level is to a lesser extent likely related to body Mg content because it represents only 0.3% [11]. Since there is no functional biomarker for Mg status, serum Ca/Mg ratios can assess its homeostasis and measurement [30]. Ca and Mg physiology helps to comprehend the potential impact of the Ca/Mg ratio, since one is physiologically antagonistic to the other [13]. Therefore, the cellular Ca/Mg ratio is crucial for Ca-dependent signaling events, making it possible that cytosolic Ca activation results from Mg deficiency [13,157]. The increase in the Ca/Mg ratio at the cellular level is a potentially deleterious factor and contributes to arteriosclerosis and HTN development [158]. An imbalance of these nutrients in the blood can lead to NCDs, including MetS, DM, HTN, and CAD. Serum Mg deficiency or high serum Ca levels may lead to an increase in heart failure and CVD risk [29], hyperlipidemia, HTN, DM, and obesity [137] due to abnormal reactions of neutrophils, lymphocytes and monocytes/macrophages, vascular smooth muscle cells and vascular endothelial cells, and the accumulation of cholesterol ester in the arterial wall [137]. In the cell membranes and lymphocytes of HTN patients, a significant increase in Ca, a decrease in Mg, and a Ca/Mg ratio > 2 were observed. Lack of Mg increases the risk for lipid peroxidation and the development of dyslipoproteinemia [159].

Low serum Mg levels and high Ca/Mg ratios may be independent factors for predicting all-cause and CVD mortality in CAD patients [59]. Yuan et al. described an augmented Mg and a reduced Ca/Mg ratio in whole blood associated with MetS [160]. Other experimental data suggest that a higher intracellular Ca/Mg ratio, induced by a diet elevated in Ca and deficient in Mg, may cause HTN, IR, and MetS [161]. Women with preeclampsia had a meaningfully higher mean serum Ca/Mg ratio than women without preeclampsia [162]. Moreover, the Ca/Mg ratio plays the main role in the stimulation and transmission of nerve cell signals. A reduction in serum Ca/Mg ratio can increase excitability and cause burst firing, long-term potentiation, pain transmission, epileptogenesis, and nerve damage [163]. According to a study of healthy Chinese women of childbearing age (18–44 years), a reference range of 2.41 to 3.44 for serum Ca/Mg ratio was suggested [164]. Li et al. point out that a moderate Ca/Mg ratio (3.91–4.70) had the lowest mortality risk [59].

Emerging evidence suggests that the serum Mg/Ca quotient is a more practical and sensitive indicator of Mg status and/or turnover, than the serum magnesium level alone [11,60]. In our study, the mean serum Ca/Mg ratio (4.79) was high, and no significant difference between groups by BMI was found. Based on the cutoff > 4.70, 54% (42 patients) of our series had a high serum Ca/Mg ratio and 4% (3 cases) had a low ratio (<3.91). Only three patients had low serum Ca/Mg ratio (<3.91) and low Ca/Mg intake ratio (<1.70). Only three subjects had regular Ca/Mg ratios. With a serum Mg/Ca ratio of 0.4, the optimal and the range from 0.36 to 0.28 was too low; the serum Mg/Ca ratio of our results of 0.21 was very low, and 49% (38 cases) of patients were below 0.20. Data from 1064 patients aged 60 years and hospitalized by COVID-19 show that an Mg/Ca ratio ≤ 0.20 is powerfully related with mortality in patients with severe COVID-19 [165]. The Mg/Ca ratio ≤ 0.20 is used as a mortality predictor in patients with CV and neoplastic illnesses [165,166]. These results support the high risk in our series of developing chronic diseases and an increased risk of mortality.

On the other hand, the Ca/Mg intake ratio is a biomarker of interest for moderating chronic disease [28]. The optimal dietary Ca/Mg intake ratio is close to 2.00 [126]. It is noteworthy that an excessive Ca intake may negatively affect Mg absorption, increasing Mg requirements and leading to subclinical Mg deficiency [12]. In our study, the mean dietary Ca/Mg intake ratio (1.06 ± 0.51) was low (<1.70), and there was no significant difference between groups by BMI, and 90% of this series (70 cases) had a low Ca/Mg intake ratio < 1.70 and 3% (2 subjects) had a high ratio (>2.60). Out of 33 patients with normal serum Ca/Mg ratio, 25 subjects had low, and 1 case had a high Ca/Mg intake ratio. Of the 42 patients with high serum Ca/Mg ratio, 40 participants had a low Ca/Mg intake ratio. Hibler et al. reported that increased physical activity along with a dietary Ca/Mg intake ratio between 1.70 and 2.60 showed a reduced risk of cancer death [167]. In two large cohort studies led in Chinese populations with low Ca/Mg intake ratios (mean 1.70), this ratio significantly modified mortality risk [166]. Several studies described that this ratio is associated with all-cause mortality and the incidence of acute myocardial infarction [168], being a useful biomarker for its diagnosis [169]. The Ca/Mg intake ratio is a significant accurate marker of CVD and all-cause mortality in patients starting dialysis. It is clinically uncertain whether an increased Mg and/or reduced Ca level improves CVD mortality risk [30]. Therefore, based on the Ca/Mg intake ratio, our series had a high risk of developing NCDs.

Inadequate Mg intakes along with elevated Ca intakes and high Ca/Mg intake ratios (>2.60) can increase CVD and MetS risk [161], colorectal cancer [170], prostate cancer [171], breast cancer [172], and cancer mortality [173], as well as altered vitamin D status [174]. An elevated Mg intake was meaningfully associated with a reduced risk in colorectal adenoma, but only with dietary Ca/Mg ratios ≤ 2.78. In the prostate, lung, colorectal, and ovarian (PLCO) cancer screening trial [175], patients with elevated Ca consumption and Ca/Mg intake ratios between 1.70 and 2.50 had a reduced risk of new cancers [13]. Data from the North Carolina–Louisiana Prostate Cancer Project [171] reported that both African-American and European-American men with prostate cancer and a Ca/Mg intake ratio > 2.50 were more likely to have highly aggressive prostate cancer. Nevertheless, in women with breast cancer, postmenopausal women with the highest Ca/Mg ratio > 2.59, had a significantly lower risk of all-cause cancer [172]. Furthermore, elevated Mg intake was associated with a reduced risk of noncardiac gastric carcinoma regardless of the Ca/Mg ratio. However, in patients with a Ca/Mg intake ratio < 1.70, there was an increased risk of esophageal adenocarcinoma [176]. Hence, our series also has a high risk of developing cancers.

### 4.8. Risk of Other Chronic Illnesses

It is important to bear in mind that few studies have discussed the association between Mg status and metabolic disorders in childhood and adolescence with chronic illnesses. Even though previous studies have shown that 40% to 60% of PICU patients have hypomagnesemia [177], in 974 critically ill children with sepsis, after controlling for confounding factors (acute KD and Ca and K metabolic disturbances), hypermagnesemia (2.43 mg/dL or 1 mmol/L) was the only factor associated with increased mortality. Suggesting that serum Mg may be a useful prognostic biomarker for children with sepsis [178]. In a study performed on 3954 apparently healthy Mexican children without any T2D, hepatic, renal, or endocrine disease, results indicated that the serum Mg level < 1.8 mg/dL (0.74 mmol/dL) was significantly associated with preHTN and HTN [179]. In 20,119 patients (meta-analyses) of HTN, an inverse association was found between patients with high Mg (>300 mg) consumption compared to those with a deficient Mg (<200 mg) intake [180]. Seventy-two percent of children with ADHD had Mg deficiency [181]. In a study, there were significantly lower serum Mg levels in 25 children on regular HD than in the controls (1.7 mg/dL or 0.69 mmol/dL vs. 2.31 mg/dL or 0.95 mmol/dL), in which structural abnormalities in the great vessels were associated with low serum Mg levels [182]. Hypomagnesemia in children with malignant neoplasms is seen especially with certain medications and can be complicated by diarrhea and malnutrition. In severe hypomagnesemia cases, it can cause alterations in the neuromuscular and cardiovascular systems [183].

Chronic reduced intake of Mg leads to a deficiency of Mg in serum and intracellular levels, principally evident in obese individuals with MetS, the elderly, and nonwhite people with IR [184]. However, the association between Mg deficit and IR is described also in childhood [185]. Mg deficiency is more prevalent in subjects with body BMI in the obese range than in the normal American population [186]. Deficient Mg status in obese children may be secondary to a decreased dietary Mg intake [91]. Furthermore, elevated serum Ca levels are associated with glucose metabolism, such as fasting glucose, glucose intolerance, IR, β-cell disfunction, and T2D [187]. In our series, CT and serum and dietary Mg in obese patients had no significant differences compared to non-obese subjects, and HDL-cholesterol was higher in eutrophic patients than in underfed and obese ones. In contrast, other studies in obese children show reduced serum Mg compared to the healthy control group. Furthermore, Mg levels are inversely associated with the degree of obesity and are connected to an unfavorable serum lipid profile and elevated systemic blood pressure compared to healthy controls [91,185,188]. Mg supplement or augmented ingestion of Mg-rich foods to correct its deficiency may represent an essential and inexpensive tool in preventing T2D in obese children [189].

In our series, CV risk index was associated with serum Mg (R^2^ = 0.119) and Ca/Mg ratio (R^2^ = 0.109). In the undernutrition group with serum Mg (R^2^ = 0.176), and Ca/Mg ratio (R^2^ = 0.200), and in the eutrophic group with serum Ca/Mg ratio (R^2^ = 0.261), and diet Mg intake (R^2^ = 0.210). Low Mg intakes raise inflammatory and CVD risks and increasing dietary Mg intake is associated with a reduced risk of stroke, heart failure, DM, and all-cause mortality [190]. Of all-cause and CVD mortality in patients with CAD, patients with low serum Mg levels (0.94–1.97 mg/dL or 0.38–1.97 mmol/dL) had the highest mortality risk compared with moderate serum Mg. High serum Mg levels (2.33–5.54 mg/dL or 0.95–2.28 mmol/dL) were associated with higher CAD mortality [59]. Hypomagnesemia is associated with abnormal platelet aggregation, coagulation abnormalities [191], endothelial dysfunction [192], and myocardial damage [193], in severely ill COVID-19 patients [194]. Mg supplement can help to control blood pressure and reduce the CV risk factors associated with HTN, especially in hypertensive individuals [195]. Mg’s actions as an antihypertensive, antidysrhythmic, anti-inflammatory, and anticoagulant agent can be of benefit in the prevention and treatment of CVD [84,196]. A deeper study and analysis of the associations shown in this manuscript may be crucial knowledge to better understand the metabolism of these nutrients.

### 4.9. Therapeutic Measures

Studies have shown a reduced risk of various chronic illnesses with increased Mg intake or supplementation [57]. The potential beneficial effect of Mg intake on chronic diseases may be, at least in part, explained by inhibiting inflammation [131]. It is evident that Mg effectively treats respiratory diseases such as asthma and pneumonia because of its anti-inflammatory, antioxidant, and smooth muscle relaxant properties [197]. Despite the lack of agreement, an appropriate dietary pattern, including the right intake of Mg, improves MetS by reducing blood pressure, hyperglycemia, and hypertriglyceridemia [189]. It was recently suggested that high-Ca diets attenuate lipid deposition in adipocytes and thus forestall weight gain [198]. The Mg therapy led to improvements in behavior, both large- and small-scale mobility, decreased the level of anxiety and aggression, increased the attention of ADHD children [199], and in preventing T2D in obese children [189]. Mg intake or Mg supplementation has a positive impact in patients with DM or depression [95], preeclampsia, migraine, and CAD [13], and acute migraine headaches [200]. Several studies have linked a high intake of Ca [201] and Mg [202] to a reduced risk of colorectal cancer or polyps.

Because of chronic diseases, medications, decreases in food crop Mg content, and the availability of refined and processed foods, most people in modern societies are at risk for Mg deficiency [82]. A healthy diet in terms of adherence to the Mediterranean diet, rich in fruits, legumes, vegetables, olive oil, herbs, spices, and high fiber intake, can help reduce the risk of NCD (obesity, DM, nonalcoholic fatty liver disease, MetS, and CV events) [203]. The intake of food rich in Mg including whole grains, nuts and seeds, legumes, and dark-green vegetables was associated with a lower incidence of obesity, T2D, and MetS [204]. All patients with malignancies and hypomagnesemia should be supplemented with Mg, and urgent treatment is indicated when serum Mg decreases below 1.0 mg/dL (0.41 mmol/dL), a level under which symptoms may develop [183]. Many experts believe that the ideal Mg intake should be based on body weight (4 to 6 mg per kg/day) [84,199]. In the treatment of Mg deficiency, organically bound Mg salts such as Mg citrate, gluconate, orotate, or aspartate are recommended due to their high bioavailability [205]. An adequate serum control of these nutrients in populations at risk and an adequate dietary balance between Ca and Mg are crucial factors that can improve the health and well-being of children and adolescents with chronic diseases and may contribute to reducing the risk of NCDs in adulthood.

At this point, we need to consider several highlights. First of all, the mean serum levels and dietary Mg and Ca intake were normal. Secondly, 45% had hypomagnesemia, 12% had hypermagnesemia, and 26% and 24% had inadequate and high Mg intake, respectively. Only 6% of patients had poor Mg intake and hypomagnesemia. Thirdly, 54% and 90% of our series had an elevated serum Ca/Mg ratio > 4.70 (mean 4.79) and a low Ca/Mg intake ratio < 1.70 (mean 1.06), respectively. Last, but not least, this study demonstrated that serum Mg and Ca levels, dietary Mg and Ca intake, serum Ca/Mg ratio, and Ca/Mg intake ratio had a meaningful association with several of the nutritional parameters studied. Regarding all the highlights, 64% of children and adolescents with chronic diseases had a high risk of Mg deficiency, and 79% were at elevated risk of having abnormal Mg status and developing other chronic sicknesses and even several cancers.

The results respond to the main aim of this study and indicate the need to continue studying the relationship between the nutritional status of patients with chronic conditions and abnormal micronutrient status to understand the essential balance between both. A limitation of this study is the small number of participants in the nutritional groups. However, its strengths lie in assessing serum and dietary Mg levels and their relationship with anthropometric, biochemical, and diet indicators. We suggest implementing multicenter trials to improve the knowledge of the Mg status of these patients and to determine the necessary and appropriate amount of Mg supplementation to improve the nutritional level in these patients when necessary, and even consider for effective prevention, delivering personalized nutritional recommendations.

## 5. Conclusions

In this series of children and adolescent patients with chronic conditions, the mean serum level and dietary Mg intake were standard. Only five patients had a serum magnesium deficiency and an inadequate magnesium intake. A total of 45% of patients had serum Mg deficiency, 12% hypermagnesemia, 26% had inadequate Mg intake, and 24% had a high Mg intake. A total of 54% of subjects had a high serum Ca/Mg ratio and 90% of patients had a low Ca/Mg intake ratio. Serum magnesium concentration and dietary magnesium intake were associated with several nutritional biomarkers. There were 64% of the children and adolescents with chronic diseases with a high risk of Mg deficiency, and 79% with an elevated risk of having abnormal Mg status and developing other chronic sicknesses and even several cancers.

## Figures and Tables

**Table 1:** Baseline characteristics of children with chronic disease by BMI groups (*n* = 78).

Characteristics	Total (*n* = 78)	Obesity (*n* = 24)	Undernutrition (*n* = 30)	Eutrophic (*n* = 24)	*p*-Value
	Mean ± SD	Mean ± SD	Mean ± SD	Mean ± SD	
Age (years)	9.6 ± 4.8	11 ± 4	7 ± 5	10 ± 5	0.003 *
Children (age in years)	6 ± 3	7 ± 3	4 ± 3	7 ± 3	0.026 *
Head circumference (cm)	52 ± 4	55 ± 3	49 ± 3	53 ± 3	0.000 *
Weight-for-age (kg)	38 ± 26	63 ± 24	18 ± 12	38 ± 18	0.000 *
Weight-for-age Z-score	38 ± 26	63 ± 24	18 ± 12	38 ± 18	0.000 *
Height-for-age (cm)	131 ± 31	147 ± 21	112 ± 30	139 ± 28	0.000 *
Height-for-age Z-score	−0.76 ± 1.5	−0.7 ± 1.3	−1.6 ± 1.6	−0.4 ± 1.2	0.000 *
Body mass index (kg/cm^2^)	19 ± 7.2	28 ± 5	13 ± 1.4	18 ± 2.8	0.000 *
Waist circumference (cm)	63 ± 18	83 ± 15	48 ± 9	62 ± 10	0.000 *
Hip circumference (cm)	76 ± 24	98 ± 17	56 ± 17	77 ± 18	0.000 *
Waist/height ratio	0.49 ± 0.09	0.57 ± 0.07	0.45 ± 0.09	0.45 ± 0.05	0.000 *
Bicipital skinfold (mm)	8.1 ± 6.9	11.1 ± 8.8	5.8 ± 4.2	7.9 ± 6.4	0.016 *
Sum of skinfolds Z-score	47 ± 36	91 ± 24	18 ± 5	38 ± 25	0.000 *
Muscle mass by anthropometry (kg)	28 ± 15	40 ± 15	16 ± 9	29 ± 12	0.000 *
Muscle mass by BIA (kg)	30 ± 16	41 ± 15	19 ± 18	29 ± 13	0.000 *
Glucose (mg/dL)	97 ± 53	98 ± 48	80 ± 13	116 ± 53	0.040 *
Creatine (mg/dL)	0.50 ± 0.19	0.52 ± 0.16	0.42 ± 0.14	0.58 ± 0.25	0.008 *
Total bilirubin (mg/dL)	0.50 ± 0.34	0.47 ± 0.17	0.39 ± 0.24	0.65 ± 0.48	0.032 *
Total cholesterol (mg/dL)	174 ± 38	161 ± 29	174 ± 40	187 ± 40	0.072
HDL-cholesterol (mg/dL)	55 ± 16	48 ± 11	56 ± 18	61 ± 17	0.027 *
Aspartate amino transferase (U/L)	28 ± 10	25 ± 9	34 ± 11	24 ± 7	0.000 *
Vitamin B 12 (pg/mL)	690 ± 276	549 ± 182	786 ± 246	708 ± 338	0.006 *
Vitamin E (µg/mL)	16 ± 6	13 ± 4	16 ± 6	17 ± 6	0.022 *
Serum magnesium (mg/dL)	2.08 ± 0.19	2.09 ± 0.19	2.1 ± 0.18	2.04 ± 0.20	0.461
Calcium (mg/dL)	9.9 ± 0.4	9.8 ± 0.4	10 ± 0.4	9.8 ± 0.4	0.118
Serum calcium/magnesium ratio	4.79 ± 0.47	4.72 ± 0.53	4.79 ± 0.44	4.86 ± 0.46	0.621
IGF-1 (ng/mL)	212 ± 136	264 ± 119	149 ± 115	241 ± 152	0.004 *
Leucocytes (cell/mm^3^)	7465 ± 2239	7025 ± 2373	8344 ± 2349	6806 ± 1593	0.020 *
Lymphocytes (cell/mm^3^)	3080 ± 1432	2887 ± 312	3606 ± 1641	2615 ± 719	0.028 *
Complement C3 (mg/dL)	114 ± 21	126 ± 16	108 ± 19	111 ± 23	0.016 *
Complement C4 (mg/dL)	21 ± 7	25 ± 6	18 ± 7	22 ± 8	0.005 *
Magnesium intake (%DRI)	105 ± 39	107 ± 41	104 ± 41	104 ± 34	0.942
Calcium intake (%DRI)	102 ± 37	94 ± 28	103 ± 34	110 ± 46	0.338
Calcium/magnesium intake ratio	1.06 ± 0.51	0.97 ± 0.45	109 ± 0.49	1.11 ± 0.57	0.555
Folic acid (%DRI)	167 ± 86	189 ± 83	133 ± 69	187 ± 96	0.024 *
Hypomagnesemia (%)	35 (45)	11 (14)	12 (15)	12 (15)	
Hypermagnesemia (%)	9 (11)	4 (5)	3 (4)	2 (2)	
Low serum Ca/Mg ratio (%)	3 (4)	2 (3)	1 (1)	0	
High serum Ca/Mg ratio (%)	42 (54)	11 (14)	18 (23)	13 (17)	
Deficient magnesium intake (%)	20 (26)	6 (8)	9 (12)	5 (6)	
Deficient calcium intake (%)	27 (35)	11 (14)	7 (9)	9 (12)	
High magnesium intake (%)	19 (24)	7 (9)	7 (9)	5 (6)	
High calcium intake	22 (28)	4 (5)	7 (9)	11 (14)	
Low Ca/Mg intake ratio (%)	70 (90)	22 (28)	25 (32)	23 (30)	
High Ca/Mg intake ratio (%)	2 (2)	1 (1)	0	1 (1)	

Legend: % DRI: percentage of dietary reference intake (normal values 80–120%DRI). * *p*-value < 0.05.

**Table 2:** Number and percentage of cases with deficient/adequate magnesium intake versus normal/abnormal serum magnesium levels. (*n* = 78).

Serum Magnesium Concentration	Dietary Magnesium Intake (*n*, %)			
	Deficiency	Normal	High	Total
Asymptomatic hypomagnesemia	1 (1.4)	4 (5.1)	3 (3.8)	8 (10.3)
Chronic latent magnesium deficiency	4 (5.1)	14 (18)	9 (11.5)	27 (34.6)
Normal levels	11 (14.1)	16 (20.5)	7 (9)	34 (43.6)
Asymptomatic hypermagnesemia	4 (5.1)	5 (6.4)	0	9 (11.5)
Total	20 (25.7)	39 (50)	19 (24.3)	78 (100)

**Table 3:** Number and percentage of cases with deficient/adequate calcium/magnesium intake ratio versus normal/abnormal serum calcium/magnesium ratio (*n* = 78).

Serum Calcium/Magnesium Ratio	Dietary Calcium/Magnesium Intake Ratio (*n*, %)			
	Low	Normal	High	Total
Low	3 (3.8)	0	0	3 (3.8)
Normal	27 (34.7)	3 (3.8)	3 (3.8)	33 (42.3)
High	40 (51.3)	2 (2.6)	0	42 (53.9)
Total	70 (89.8)	5 (6.4)	3 (3.8)	78

**Table 4:** Basic characteristic of patients with hypermagnesemia (*n* = 9).

Cases	1	2	3	4	5	6	7	8	9
Gender	Female	Female	Male	Female	Male	Female	Male	Female	Female
Age (years)	1	1	2	12	13	13	13	13	16
Nutritional status by BMI	under	under	normal	under	obese	obese	obese	normal	obese
Calcium intake (% DRI)		57			79	71	78	73	64
Magnesium intake (% DRI)	108	80	107	58	75	89	84	78	78
Zinc intake (% DRI)		22	31	45	55	74	74	58	48
Iodine intake (%DRI)	55	31	54	45	68	55	47	41	
Cholesterol (mg/dL)								289	
LDL-cholesterol (mg/dL)			119					197	131
Serum Mg (mg/dL)	2.4	2.4	2.4	2.5	2.4	2.5	2.4	2.4	2.4
Serum zinc (µg/dL)						69	61		
Serum copper (µg/dL)			175						
High Cu/Zn ratio			2.3						
Leucocytes (cell/mm^3^)							750		
Lymphocytes (cell/mm^3^)							490		
ESR (mm/h)			26			37	36		

Legend: BMI: body mass index. % DRI: percentage of dietary reference intake.

**Table 5:** Differences between participants with chronic diseases (*n* = 78).

Characteristics	Children < 5 y		Children ≥ 5 y	*p*-Value
Serum magnesium level (mg/dL)	2.15 ± 0.18		2.05 ± 0.19	0.036 *
Serum calcium level (mg/dL)	10.1 ± 0.5		9.8 ± 0.4	0.021 *
Dietary calcium intake (%DRI)	115 ± 39		96 ± 34	0.044 *
**Age Group**	**Children**		**Adolescents**	
Dietary magnesium intake (%DRI)	120 ± 45		87 ± 19	0.000 *
Serum calcium level (mg/dL)	10 ± 0.4		9.8 ± 0.4	0.015 *
Dietary calcium intake (%DRI)	119 ± 36		83 ± 27	0.000 *
**Erythrocyte Sedimentation Rate**	**Normal**		**High**	
Serum magnesium level (mg/dL)	2.06 ± 0.19		2.18 ± 0.15	0.024 *
Serum calcium level (mg/dL)	9.9 ± 0.4		10.1 ± 0.4	0.045 *
**Serum magnesium level**	**Low**	**Normal**	**High**	
Dietary magnesium intake (%DRI)	113 ± 34	-	84 ± 16	0.001 *
	-	106 ± 44	84 ± 16	0.032 *
Serum magnesium (mg/dL)	1.91 ± 0.85	2.18 ± 0.76	2.42 ± 0.4	0.000 *
Serum calcium/magnesium ratio	5.19 ± 0.19	4.53 ± 0.22	4.12 ± 0.31	0.000 *
**Dietary magnesium intake**	**Low**	**Normal**	**High**	
Serum magnesium level (mg/dL)	2.18 ± 0.19	2.08 ± 0.19	2.00 ± 0.15	0.018 *
Dietary calcium intake (%DRI)	84 ± 39	100 ± 34	123 ± 28	0.003 *
Dietary magnesium intake (%DRI)	64 ± 15	99 ± 11	159 ± 27	0.000 *
Serum calcium/magnesium ratio	4.62 ± 0.47	4.77 ± 0.45	5.02 ± 0.39	0.027 *
Calcium/magnesium intake ratio	1.39 ± 0.73	1.02 ± 0.36	0.80 ± 0.24	0.001 *

Legend: %DRI: percentage of dietary reference intake. * *p* < 0.05.

**Table 6:** Significant Odds Ratios throughout the series (*n* = 78).

	Fisher’s Exact Test	Odds Ratio	95% Confidence Interval		Cochran’s	Mantel–Haenszel
			Lower	Upper		
**Dietary magnesium deficiency**						
Microcephaly	0.030	3.556	1.133	11.154	0.025	0.055
Children under 10-year-old	0.015	1.397	1.045	1.866	0.015	0.032
Deficient energy intake	0.011	4.182	1.398	12.512	0.008	0.019
Deficient total fat intake	0.019	4.083	1.273	13.100	0.014	0.033
Deficient calcium intake	0.008	4.200	1.439	12.262	0.007	0.015
High vitamin B1 intake	0.004	5.844	1.789	19.094	0.002	0.006
High vitamin B2 intake	0.017	3.750	1.269	11.080	0.014	0.030
High niacin intake	0.000	7.897	2.528	24.670	0.000	0.000
High iron intake	0.027	3.184	1.098	9.234	0.029	0.056
**High dietary magnesium intake**						
Deficient vitamin E intake	0.027	3.257	1.108	9.572	0.028	0.055
Deficient zinc intake	0.001	6.571	2.127	20.304	0.001	0.002
Deficient iodine intake	0.010	4.259	1.415	12.822	0.007	0.018
High calcium intake	0.010	4.259	1.415	12.822	0.007	0.018

**Table 7:** Association between bone conduction speed (BCS) through bioelectrical impedance analysis (BIA) with anthropometric parameters (*n* = 78) [42].

	Total Series		Obesity		Undernutrition		Eutrophic	
	*r*	*p*-Value	*r*	*p*-Value	*r*	*p*-Value	*r*	*p*-Value
Age (months)	0.773 **	0.000	0.761 **	0.000	0.732 **	0.000	0.876 **	0.000
Age-for-50° height	0.638 **		0.539 *	0.012	0.799 **		0.639 **	0.001
Weight-for-age	0.525 **		0.589 **	0.005	0.786 **		0.834 **	0.000
Height-for-age	0.742 **		0.700 **	0.000	0.791 **		0.834 **	
Weight-for-height	0.287 *	0.016	-	-	-	-	0.576 **	0.004
Body mass index	0.261 *	0.029	-	-	-	-	0.626 **	0.001
Muscle mass by A. (kg)	0.622 **	0.000	0.653 **	0.001	0.765 **	0.000	0.889 **	0.000
Fat mass by A. (kg)	0.354 **	0.003	0.453 *	0.039	0.737 **		0.598 **	0.003
Muscle mass by BIA	0.490 **	0.000	0.492 *	0.023	-	-	0.871 **	0.000
Fat mass by BIA	0.330 **	0.008	0.572 **	0.007	-	-	0.561 **	0.005

Legend: A: Anthropometry. BIA: bioelectrical impedance analysis. * *p* < 0.05. ** *p* < 0.01 (2-tailed).

**Table 8:** Regression analysis between serum level and dietary intake of magnesium (Mg) and calcium (Ca), serum and dietary Ca/Mg ratios, and nutritional parameters (*n* = 78).

Serum Magnesium	Serum Calcium	Serum Ca/Mg Ratio	Magnesium Intake	Calcium Intake	Ca/Mg Intake Ratio
Linear	regression	analyses			
	*r* = 0.135, *p* = 0.001 Age in years			*r* = 0.089, *p* = 0.008 Age in years	
	*r* = 0.125, *p* = 0.005 Head circumference	*r* = 0.066, *p* = 0.047 Arm perimeter	*r* = 0.157, *p* = 0.001 Height-for-age	*r* = 0.143, *p* = 0.002 Height-for-age	
	*r* = 0.141, *p* = 0.008 Fat mass by BIA		*r* = 0.110, *p* = 0.022 kg mass muscular by BIA		
*r* = 0.071, *p* = 0.023 Vitamin E intake					
	*r* = 0.081, *p* = 0.017 BCS absolute value		*r* = 0.057, *p* = 0.048 BCS absolute value		
	*r* = 0.062, *p* = 0.043 Serum vitamin C	*r* = 0.138, *p* = 0.007 HDL-cholesterol	*r* = 0.059, *p* = 0.039 IGF-1	*r* = 0.223, *p* = 0.000 Serum vitamin B12	*r* = 0.091, *p* = 0.013 Serum vitamin B12
	*r* = 0255, *p* = 0.000 Serum phosphorus	*r* = 0.046, *p* = 0.038 Serum magnesium		*r* = 0.062, *p* = 0.033 Serum phosphorus	
*r* = 0.087, *p* = 0.016 MCH	*r* = 0.214, *p* = 0.000 Lymphocytes	*r* = 0.121, *p* = 0.004 MCH	*r* = 0.087, *p* = 0.030 IgA	*r* = 0.124, *p* = 0.003 Lymphocytes	*r* = 0.089, *p* = 0.029 T-lymphocytes CD16+56
*r* = 0.119, *p* = 0.003 CV risk index		*r* = 0.109, *p* = 0.005 CV risk index			
Multilinear	regression	analyses			
			*r* = 0.650, *p* = 0.000 Energy, vitamin B12, folic acid, zinc, vitamin E and cholesterol intake	*r* = 0.274, *p* = 0.000 Energy, proteins, and iron intake	*r* = 0.391, *p* = 0.000 Magnesium, niacin, iron, and protein intake
*r* = 0.160, *p* = 0.004 Serum vitamin D, and beta-carotene					
*r* = 0.298, *p* = 0.001 HDL-cholesterol, total bilirubin, and LDL-cholesterol	*r* = 0.466, *p* = 0.000 Creatinine, albumin, alkaline phosphatase, and ESR	*r* = 0.990, *p* = 0.000 Serum Mg, Ca, and copper			
*r* = 0.989, *p* = 0.000 Serum Ca/Mg ratio, calcium, and copper			*r* = 0.141, *p* = 0.005 Serum phosphorus, and Mg		
*r* = 0.249, *p* = 0.004 Complement C4, IgG3, and CD4/CD8 ratio		*r* = 0.269, *p* = 0.001 T-lymphocytes C4 and IgG3	*r* = 0.188, *p* = 0.005 Transferrin, and BUN	*r* = 0.287, *p* = 0.001 T-lymphocytes CD16+56, CD4/CD8 ratio, and IgA	*r* = 0.162, *p* = 0.003 MVC, and platelets

Legend: BIA: bioelectrical impedance analysis. BCS: Bone conduction speed. MCH: Mean corpuscular hemoglobin. IGF-1: Insulin-like growth factor-1. CV: Cardiovascular. ESR: Erythrocyte sedimentation rate. MCH: Mean corpuscular hemoglobin. MCV: Mean corpuscular volume. BUN: Blood urea nitrogen.

## Supplementary Materials

The following supporting information can be downloaded at: https://www.mdpi.com/article/10.3390/nu14142941/s1, Table S1: Regression analysis between serum magnesium, dietary magnesium intake, and nutritional parameters in the groups by body mass index (*n* = 78); Table S2: Regression analysis between serum calcium, dietary calcium intake, and nutritional parameters in the groups by body mass index (*n* = 78). Table S3: Regression analysis between serum calcium/magnesium and calcium/magnesium intake ratios and nutritional parameters in the groups by body mass index (*n* = 78); Table S4. Regression analysis between serum and dietary calcium and magnesium levels and calcium/magnesium ratios and nutritional parameters in children (*n* = 42); Table S5. Regression analysis between serum and dietary calcium and magnesium levels and calcium/magnesium ratios and nutritional parameters in adolescents (*n* = 36); Table S6: Basic characteristics of patients with low bone conduction speed; Figure S1: Regression serum calcium (mg/dL) by age (years); Figure S2: Regression dietary calcium intake (%Dietary Reference Intake) by age (years).
