# Supplementary material for: Magnesium Status and Ca/Mg Ratios in a Series of Children and Adolescents with Chronic Diseases
Source: Nutrients. 2022 Jul 18;14(14):2941. doi: 10.3390/nu14142941 (PMC9315923; doi:10.3390/nu14142941)
Supplement: Supplementary file 1 [file nutrients-14-02941-s001.zip › nutrients-1771391-supplementary.pdf]

Magnesium Status and Ca/Mg ratios in a Series of Children and Adolescents with Chronic Diseases

Marlene Fabiola Escobedo-Monge [1,*], Enrique Barrado [2], Joaquín Parodi-Román [3], María Antonieta Escobedo-Monge [4] and José Manuel Marugán-Miguelsanz [5]

Table S1. Regression analysis between serum magnesium, dietary magnesium intake and nutritional parameters in the groups by body mass index ($n = 78$).

**Obesity ($n = 24$)**		**Undernutrition ($n = 30$)**		**Eutrophic ($n = 24$)**	
**Serum magnesium**	**Magnesium intake**	**Serum magnesium**	**Magnesium intake**	**Serum magnesium**	**Magnesium intake**
Linear	regression	analyses			
	$r = 0.347$, $p = 0.002$ Height-for-age	$r = 0.137$, $p = 0.048$ Height-for-age	$r = 0.236$, $p = 0.007$ Weight-for-height Z-score	$r = 0.281$, $p = 0.011$ Height-for-age Z-score	
	$r = 0.342$, $p = 0.005$ kilograms muscular mass by A.	$r = 0.263$, $p = 0.021$ BMI Z-score		$r = 0.225$, $p = 0.030$ Weight-for-age Z-score	
	$r = 0.308$, $p = 0.009$ Waist circumference	$r = 0.354$, $p = 0.002$ Waist/height ratio		$r = 0.298$, $p = 0.009$ Head circumference	$r = 0.187$, $p = 0.035$ Waist/hip ratio
$r = 0.177$, $p = 0.046$ Polyunsaturated fat intake	$r = 0.368$, $p = 0.004$ BCS absolute value				
	$r = 0.350$, $p = 0.003$ Serum vitamin B12		$r = 0.243$, $p = 0.014$ Serum vitamin B12	$r = 0.274$, $p = 0.026$ Serum vitamin D	
$r = 0.193$, $p = 0.036$ Serum zinc	$r = 0.279$, $p = 0.010$ Serum phosphorus	$r = 0.186$, $p = 0.020$ Serum phosphorus	$r = 0.292$, $p = 0.014$ BUN		$r = 0.165$, $p = 0.049$ Leucocytes
$r = 0.498$, $p = 0.000$ MCH	$r = 0.309$, $p = 0.006$ IGF-1	$r = 0.176$, $p = 0.026$ CV risk index			$r = 0.210$, $p = 0.032$ CV risk index
Multilinear	regression	analyses			
			$r = 0.438$, $p = 0.007$ Sum of skinfolds Z-score, waist/hip ratio		$r = 0.471$, $p = 0.002$ Bicipital skinfold and waist/hip ratio
	$r = 0.824$, $p = 0.000$ Calcium, monosaturated fats, protein, folic acid, and cholesterol intakes		$r = 0.759$, $p = 0.000$ Vitamin B12, and zinc intakes	$r = 0.407$, $p = 0.006$ cholesterol, carbohydrates, and vitamin B1 intake	$r = 0.696$, $p = 0.000$ energy and iodine intakes
		$r = 0.631$, $p = 0.000$ AST and glucose			
$r = 0.814$, $p = 0.001$ T-lymphocytes CD4 and Ig A	$r = 0.942$, $p = 0.000$ Ig A, complement C3 and Ig G2	$r = 0.335$, $p = 0.014$ Basophiles and leucocytes	$r = 0.563$, $p = 0.000$ Complement C3 and CD4/CD8 ratio		

Legend: A: Anthropometry. BMI: Body mass index. BCS: Bone conduction speed. BUN: Blood urea nitrogen. MCH: Mean corpuscular hemoglobin. IGF-1: Insulin-like growth factor-1. CV: Cardiovascular. AST: Aspartate aminotransferase. Ig: Immunoglobulin.

Table S2. Regression analysis between serum calcium, dietary calcium intake and nutritional parameters in the groups by body mass index (n = 78).

**Obesity (n = 24)**		**Undernutrition (n= 30)**		**Eutrophic (n = 24)**	
**Serum calcium**	**Calcium intake**	**Serum calcium**	**Calcium intake**	**Serum calcium**	**Calcium intake**
Linear	regression	analyses			
r = 0.328, p = 0.010 Subscapular skinfold Z-score			r = 0.145, p = 0.041 BMI Z-score	r = 0.182, p = 0.042 Head circumference	r = 0.173, p = 0.043 Hip circumference
	r = 0.375, p = 0.003 Fat mass by BIA		r = 0.213, p = 0.014 FA/MA index		
			r = 0.418, p = 0.000 Iron intake		r = 0.440, p = 0.000 Saturated fat intake
r = 0.220, p = 0.032 BCS absolute value					
r = 0.201, p = 0.032 Beta-carotene	r = 0.271, p = 0.011 Serum vitamin B12		r = 0.185, p = 0.036 Serum vitamin B12	r = 0.225, p = 0.040 Serum vitamin B12	
	r = 0.211, p = 0.027 Serum phosphorus	r = 0.417, p = 0.000 Serum phosphorus	r = 0.154, p = 0.039 Serum phosphorus	r = 0.199, p = 0.037 Serum zinc	
r = 0.233, p = 0.046 Creatinine	r = 0.357, p = 0.024 AST				r = 0.318, p = 0.012 T-lymphocytes CD16+56
r = 0.400, p = 0.002 Lymphocytes	r = 0.191, p = 0.042 Lymphocytes		r = 0.06, p = 0.005 Lymphocytes		r = 0.178, p = 0.040 MCHC
Multilinear	regression	analyses			
	r = 0.391, p = 0.006 Height-for-age, bicipital skinfold		r = 0.705, p = 0.000 Sum of skinfold, BMI Z-score, height-for-age Z-score, head circumference		
	r = 0.734, p = 0.000 Magnesium, vitamin B2, iron and vitamin B6		r = 0.460, p = 0.005 BUN, and HDL-cholesterol	r = 0.979, p = 0.000 Glucose, CRP, creatinine, albumin, transferrin, GGT, triglycerides, and ferritin	
r = 0.615, p = 0.014 T-lymphocytes CD8, and CD19	r = 0.673, p = 0.007 IgA, and complement C3	r = 0.523, p = 0.001 CD4/CD8 ratio, T-lymphocytes CD4			

Legend: A: BMI: Body mass index. BCS: Bone conduction speed. BIA: bioelectrical impedance analysis. FA/MA: Fat area/muscle area. AST: Aspartate amino transferase. MCHC: Mean corpuscular hemoglobin concentration. Ig: Immunoglobulin.

Table S3. Regression analysis between serum calcium/magnesium and calcium/magnesium intake ratios and nutritional parameters in the groups by body mass index ($n = 78$).

**Obesity ($n = 24$)**		**Undernutrition (n= 30)**		**Eutrophic ($n = 24$)**	
**Serum Ca/Mg ratio**	**Ca/Mg intake ratio**	**Serum Ca/Mg ratio**	**Ca/Mg intake ratio**	**Serum Ca/Mg ratio**	**Ca/Mg intake ratio**
Linear	regression	analyses			
$r = 0.191$, $p = 0.042$ Height-for-age Z-score			$r = 0.406$, $p = 0.000$ Weight-for-height Z-score		$r = 0.182$, $p = 0.038$ Waist circumference Z-score
$r = 0.237$, $p = 0.035$ Head circumference	$r = 0.270$, $p = 0.011$ IGFBP3	$r = 0.277$, $p = 0.017$ BMI Z-score	$r = 0.242$, $p = 0.012$ Waist/height ratio		$r = 0.151$, $p = 0.038$ Waist circumference
$r = 0.190$, $p = 0.049$ BCS absolute value		$r = 0.165$, $p = 0.032$ Monosaturated fat intake			
$r = 0.529$, $p = 0.000$ MCH	$r = 0.255$, $p = 0.017$ MCV			$r = 0.243$, $p = 0.020$ Basophiles	
Multilinear	regression	analyses			
		$r = 0.374$, $p = 0.006$ Waist/height and waist circumference	$r = 0.736$, $p = 0.000$ Height-for-age Z-score, suprailiac skinfold Z-score, BMI Z-score		
	$r = 0.445$, $p = 0.002$ Magnesium and vitamin B2 intake		$r = 0.529$, $p = 0.000$ Magnesium and iron intake	$r = 0.477$, $p = 0.002$ Cholesterol and carbohydrates intake	$r = 0.664$, $p = 0.000$ Saturated fat, and niacin intake
$r = 0.993$, $p = 0.000$ Serum Mg and Ca		$r = 0.991$, $p = 0.000$ Serum Mg, Ca, and phosphorus		$r = 0.987$, $p = 0.000$ Serum Mg and Ca	$r = 0.537$, $p = 0.001$ Serum vitamin B12 and C
	r = 0.576, p = 0.009 Cholesterol and prealbumin	$r = 0.694$, $p = 0.000$ HDL-cholesterol, GGT, and creatinine	$r = 0.632$, $p = 0.001$ ESR, transferrin, and GGT	$r = 0.778$, $p = 0.000$ LDL-cholesterol, total bilirubin, cholesterol, and alkaline phosphatase	
		$r = 0.405$, $p = 0.004$ MCH, and monocytes	$r = 0.673$, $p = 0.000$ MCHC, monocytes, MCV, and eosinophiles		
$r = 0.860$, $p = 0.000$ IgA, T-lymphocytes CD3, complement C4		$r = 0.820$, $p = 0.000$ IgG2, IgG4, complement C3, and IgG1	$r = 0.720$, $p = 0.000$ Complement C3, T-lymphocytes CD16+56 and CD3	$r = 0.651$, $p = 0.000$ Complement C4, IgG3, and complement C3	$r = 0.528$, $p = 0.002$ T-lymphocytes CD19 and IgG2
		$r = 0.200$, $p = 0.017$ CV risk index		$r = 0.261$, $p = 0.015$ CV risk index	

Legend: IGFBP3: insulin-like growth factor-binding protein 3. BMI: Body mass index BCS: Bone conduction speed. MCV: Mean corpuscular volume. GGT: Gamma glutamyl transpeptidase. ESR: Erythrocyte sedimentation rate. MCH: Mean corpuscular hemoglobin. MCHC: Mean corpuscular hemoglobin concentration. Ig: Immunoglobulin. CV: Cardiovascular.

Table S4. Regression analysis between serum and dietary calcium and magnesium levels and calcium/magnesium ratios and nutritional parameters in children (n = 42).

Dietary calcium	Dietary magnesium	Ca/Mg intake ratio	Serum calcium	Serum magnesium	Serum Ca/Mg ratio
	r = 0.297, p = 0.001 Waist/hip ratio			r = 0.155, p = 0.001 Waist/hip ratio	r = 0.234, p = 0.004 Kilograms muscle mass by A.
r = 0.345, p = 0.000 Vitamin A and B1 intake	r = 0.318, p = 0.000 Niacin, folic acid intake	r = 0.709, p = 0.000 Niacin, calcium, folic acid intake	r = 0.197, p = 0.017 Protein, vitamin E intake	r = 0.289, p = 0.003 Vitamin B1 and E intake	r = 0.280, p = 0.003 Vitamin B1, saturated fat intake
			r = 0.445, p = 0.001 Total protein, AST, ferritin	r = 0.397, p = 0.001 HDL-cholesterol total bilirubin	r = 0.529, p = 0.000 HDL-cholesterol, total protein, and bilirubin
r = 0.237, p = 0.004 Serum vitamin B12		r = 0.233, p = 0.004 Serum vitamin B12	r = 0.145, p = 0.026 Serum magnesium	r = 0.450, p = 0.000 Beta-carotene, serum vitamin D, calcium	r = 0.905, p = 0.000 Serum magnesium, calcium, beta-carotene
				r = 0.503, p = 0.002 IGF-1	r = 0.270, p = 0.001 IGFBP3
r = 0.133, p = 0.026 Eosinophils	r = 0.112, p = 0.043 Leucocytes				
r = 0.327, p = 0.005 T-lymphocytes CD 4, CD16+56			r = 0.298, p = 0.002 T-lymphocytes CD8	r = 0.593, p = 0.000 T-lymphocytes CD8, CD19, IgG2, IgM,	r = 0.214, p = 0.013 IgG2
				r = 0.182, p = 0.000 CV risk	r = 0.322, p = 0.001 CV risk, transferrin saturation

Legend: A: Anthropometry. AST: Aspartate aminotransferase. IGF-1: Insulin-like growth factor-1. IGFBP3: insulin-like growth factor-binding protein 3. Ig: Immunoglobulin. CV: Cardiovascular.

Table S5. Regression analysis between serum and dietary calcium and magnesium levels and calcium/magnesium ratios and nutritional parameters in adolescents (*n* = 36).

Dietary calcium	Dietary magnesium	Ca/Mg intake ratio	Serum calcium	Serum magnesium	Serum Ca/Mg ratio
r = 0.409, *p* = 0.000 Height-for-age and Waterloo II	*r* = 0.297, *p* = 0.001 Waist/hip ratio				
r = 0.448, *p* = 0.000 Magnesium, cholesterol intake	*r* = 0.448, *p* = 0.000 Calcium, vitamin D intake	*r* = 0.768, *p* = 0.000 Calcium, vitamin D, saturated fat folic acid intake -		*r* = 0.184, *p* = 0.010 Magnesium intake	*r* = 0.130, *p* = 0.033 Magnesium intake
r = 0.165, *p* = 0.032 Ferritin	*r* = 0.163, *p* = 0.033 Ferritin		*r* = 0.232, *p* = 0.011 Albumin		
	r = 0.427, *p* = 0.000 Serum vitamin D, zinc/copper ratio			*r* = 0.183, *p* = 0.016 Serum iron	*r* = 0.991, *p* = 0.000 Serum magnesium, calcium,
		r = 0.211, *p* = 0.007 Basophiles	*r* = 0.320, *p* = 0.001 Lymphocytes	*r* = 0.267, *p* = 0.002 HbCM	*r* = 0.361, *p* = 0.004 HbCM, Leucocytes
			r = 0.570, *p* = 0.000 T-lymphocytes CD8, CD19, complement C4	*r* = 0.329, *p* = 0.004 Complement C4	*r* = 0.406, *p* = 0.001 Complement C4

Legend: MCH: Mean corpuscular hemoglobin.

Table S6. Basic characteristic of patients with low bone conduction speed.

Cases No	1st	2nd	3rd	4th	5th
Age (years)	2	4	10	12	19
Gender	Male	Male	Female	Female	Female
Nutritional status by body mass index	normal	undernutrition	obesity	undernutrition	obesity
BCS Z-score	-2.31	-3.35	-2.29	-2.32	-2.24
Height Z-score	normal	normal	normal	Low	normal
Vitamin D (%DRI)	61*	15*	109	56*	17*
Calcium (%DRI)	143**	103	155**	89	109
Magnesium (%DRI)	111	92	199**	58*	116
Calcium/magnesium intake ratio	1.29*	1.11*	0.78*	1.28*	1.02*
Zinc (%DRI)	24*	96	93	45*	83
Serum vitamin D (ng/mL)	53	85**	19*	28*	10*
Serum calcium (mg/dL)	10.20	9.70	9.90	9.60	9.40
Serum magnesium (mg/dL)	2.2	2.2	1.9*	2.5**	2.1
Serum calcium/magnesium ratio	4.6	4.41	5.21**	3.84*	4.48
Serum phosphorus (mg/dL)	4.5	5.2	5.4	4.7	3.7*
Serum zinc (μg/dL)	85	76	85	85	92
Serum copper (μg/dL)	163**	143**	114	104	113
Copper/zinc ratio	1.92**	1.88**	1.34**	1.22**	1.23**
Zinc/copper ratio	0.52*	0.53*	0.75	0.82	0.81
C-reactive protein U/L	10.7**	3.75	6.3**	3.75	8.5**
Erythrocyte sedimentation rate mm/h	22**	30**	13	8	13

Legend: % DRI: percentage of dietary reference intake (normal values 80-120%DRI). Serum Ca 8.8-10.8 mg/dL [55] and hypercalcemia >11 mg/dL [56]. Symptomatic hypomagnesemia <1.22 mg/dL, asymptomatic hypomagnesemia 1.22-1.82 mg/dL, chronic latent Mg deficiency 1.82-2.07 mg/dL, interval for health 2.07-2.32 mg/dL, asymptomatic hypermagnesemia 2.32-4.86 mg/dL, symptomatic hypermagnesemia >4.86 mg/dL [57]. Deficiency vitamin D < 20 ng/mL, insufficiency vitamin D 20-30 mg/mL [61]. Serum phosphorus in children 4.5-6.5 mg/dL [55]. Hypozincemia levels <70 μg/dL in children under 10 years of age in both sexes and in females >10 years and <74 μg/dL in males older than 10 years [62]. Hypocupremia <70 μg/dL and hypercupremia >140 μg/dL [63]. The Copper/zinc ratio 0.7 to 1.0 [64]. The /copper ratio < 4.0 [65]. *Low level. **High level.

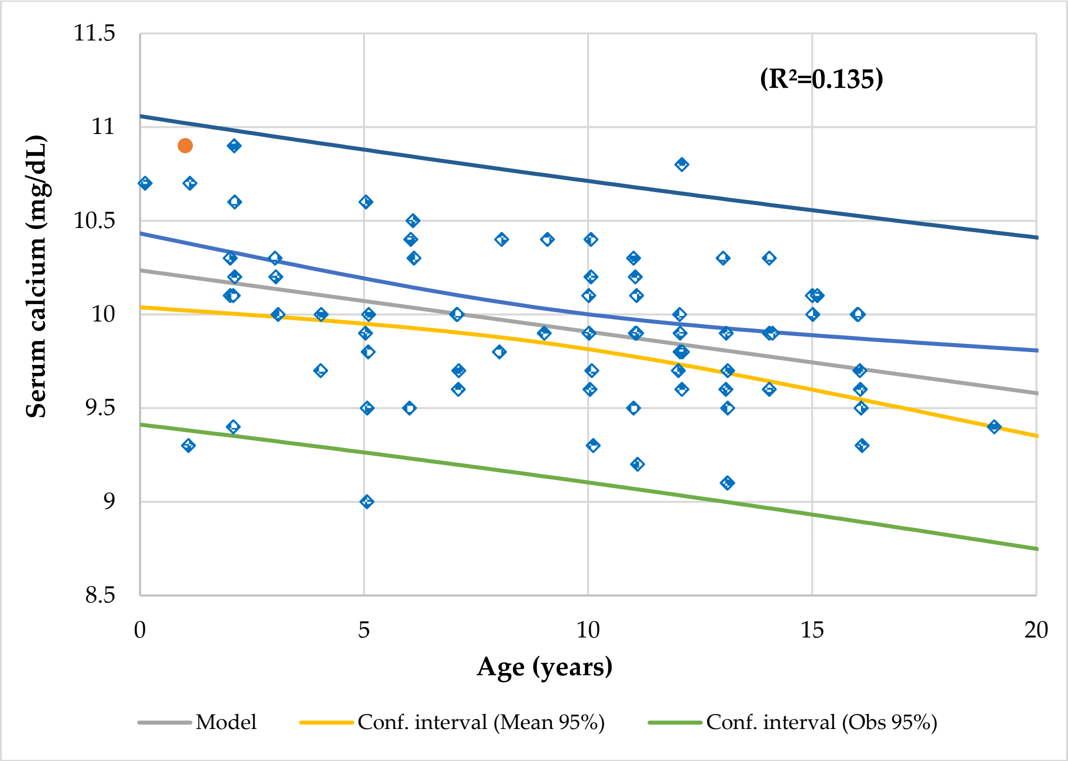

Figure S1. Regression serum calcium (mg/dL) by age (years).

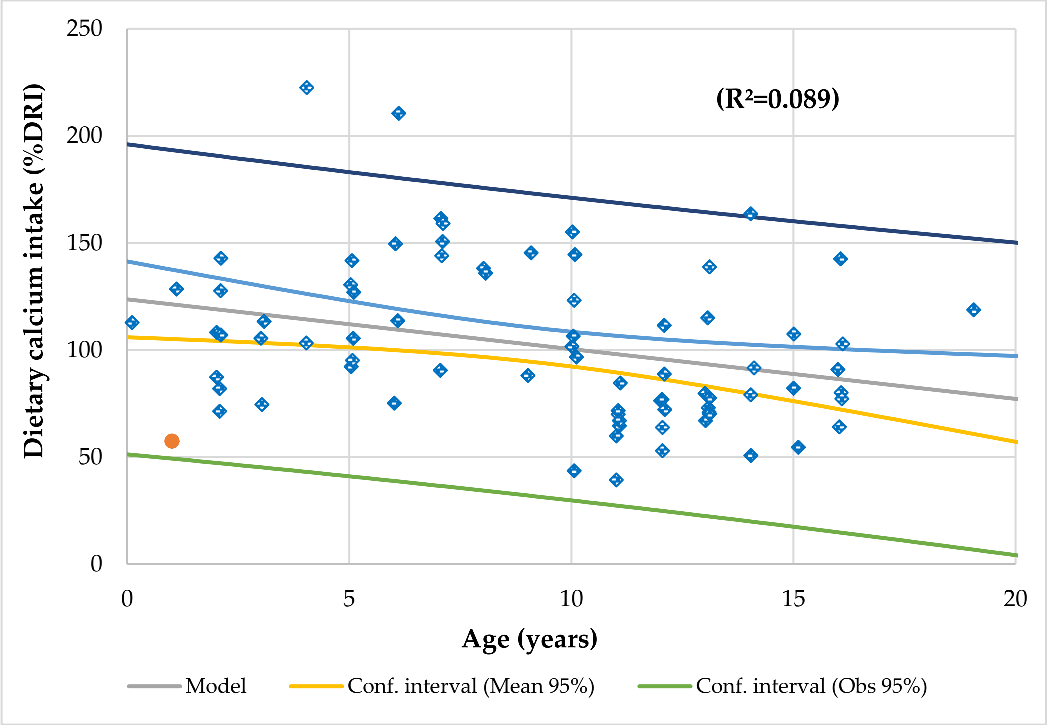

Figure S2. Regression dietary calcium intake (%Dietary Reference Intake) by age (years).
